# Supplementary material for: Engineering tumoral vascular leakiness with gold nanoparticles
Source: Nat Commun. 2023 Jul 17;14:4269. doi: 10.1038/s41467-023-40015-4 (PMC10352264; doi:10.1038/s41467-023-40015-4)
Supplement: Supplementary file 1 — Supplementary Information [file 41467_2023_40015_MOESM1_ESM.pdf]

Supplementary Materials for

Engineering Tumoral Vascular Leakiness with Gold Nanoparticles

Magdiel Inggrid Setyawati[1,2]†*, Qin Wang[3]†, Nengyi Ni[1], Jie Kai Tee[4], Katsuhiko Ariga[5,6], Pu Chun Ke[7], Han Kiat Ho[4], Yucai Wang[3]*, David Tai Leong[1]*

Correspondence to:

David Tai Leong: cheltwd@nus.edu.sg; Yucai Wang: yucaiwang@ustc.edu.cn;
Magdiel Inggrid Setyawati: misetyawati@ntu.edu.sg

This PDF file includes:

Supplementary Methods

Supplementary Figures 1 to 35

Supplementary Tables 1 to 4

Supplementary Methods

Au NPs synthesis and characterization

All glassware used for the synthesis was cleaned with aqua regia, rinsed with copious ultrapure water and dried at 60°C prior use. Au NPs with different surface roughness was synthesized following the previously reported protocol.[25] The different surface roughness was formulated with seeded-growth synthesis, whereby spherical citrate capped Au NPs were firstly synthesized and used as seed. The surface roughness was introduced on the surface of the Au NPs seed through 4-(2-hydroxyethyl)-1-piperazineethanesulfonic acid (HEPES, Sigma Aldrich) mediated branch growth.

The spherical Au NPs with the size of 13 nm were used as seeds to produce Au_{30} NPs bearing different roughness, while spherical Au NPs with the size of 18 nm were used to produce Au_{40}, Au_{60}, and Au_{70} NPs bearing different surface roughness. The spherical citrate capped Au NPs which were synthesized following the established Turkevich–Frens method.[26] Briefly, aqueous solution of chloroauric acid ($HAuCl_4$, 150mL, 0.25mM; Sigma Aldrich) was brought to boiling condition. Thereafter, aqueous solution of sodium citrate (38.75 mM; Sigma Aldrich, USA) was added to the boiling solution to initiate the reducing process. Approximately 2.9 mL and 4.8 mL of sodium citrate solution were added to produce the respective 13 nm and 18 nm Au NPs seeds. The solution was stirred and kept in the boiling condition until a red solution appeared. Thereafter, the solution was slowly cooled down in room temperature.

Au NPs with different surface roughness were produced with the presence HEPES with reducing agent hydroxylamine (NH_2OH, Sigma Aldrich). The Au_{30} NPs series was produced by adding 1.6 mL of as-synthesized 13 nm spherical Au NPs seed into 23.1 mL of HEPES aqueous solution (pH 7). Thereafter, 0.3 mL of freshly prepared NH_2OH solution (40mM) was added into the mixture and the surface roughness was achieved through addition of 5 mL of aqueous solution $HAuCl_4$ (1 mM) in dropwise manner. The reaction was maintained in stirring condition for another 2 h at room temperature to allow sufficient surface roughness growth. The different surface roughness group synthesis was achieved by controlling the working concentration of the HEPES solution. The smooth $Au_{30}R_0$ NPs were produced in the absence of HEPES solution, whereas the $Au_{30}R_1$, $Au_{30}R_2$, and $Au_{30}R_3$ were produced in the presence of 5, 25, and 50 mM HEPES,

respectively. Following the reaction, the Au_{30} NPs baring different surface roughness was dialyzed with Spectra/Por 7 (cellulose ester, MWCO 3.5 – 5 kDa; Spectrum Labs, USA) against 10 mM HEPES solution. The dialyzed Au NPs were kept in 4 ºC and further used as stock for the subsequent experiment. In the same manner, the Au_{40}, Au_{60}, and Au_{70} NPs with different surface roughness were produced by utilizing respectively 1.6, 0.8, and 0.4 mL of as-synthesized 18 nm spherical Au NPs as seeds for the surface roughness growth. The different surface roughness within these different size groups was achieved by varying the concentration of HEPES growth solution (0 – 50 mM).

The shape and the primary size of the Au NPs was determined through the field emission transmission electron microscope (FE-TEM, JEOL 2100-F). Briefly the Au NPs were dropped on carbon coated TEM grid and viewed under accelerating voltage of 200 kV. The Au NPs primary size was determined by measuring at least 50 randomly selected Au NPs with ImageJ software.[27] The Au NPs' absorbance spectra was obtained through spectra scanning from wavelength of 300 to 900 nm with microplate reader (Biotek, Singapore). Hydrodynamic size and zeta potential of the Au NPs (3.4×10^9 particles/mL) was determined through dynamic light scattering (DLS) analysis with Zetasizer (Malvern, UK). The number-based distribution was used to define the hydrodynamic size of the Au NPs. The samples were dispersed in the ultrapure water (pH = 7.2) with probe sonication (Qsonica, USA) for 1 min before the DLS analysis. To measure the NPs hydrodynamic properties in cell culture medium, the NPs were dispersed in complete EndoGRO-MV-VEGF medium with probe sonication method. Thereafter, the NPs were collected with centrifugation, re-dispersed back in ultrapure water, and analyzed with DLS.

Immunoblotting

HMVECs, which were grown in 6-wells plate, were exposed to Au_{30} NPs bearing different surface roughness (1×10^9 particles/ml). Following the exposure, the cells were washed thrice with chilled PBS and lysed with Laemmli sample buffer (63 mM Tris-HCl pH 6.8, 2% sodium dodecyl sulphate (SDS), 10% glycerol, 0.1% 2-mercaptoethanol and 0.0005% bromophenol blue). The lysis buffer was supplemented with 1% protease and phosphatase inhibitor cocktails (Sigma Aldrich). Afterward, the protein extracts were resolved on 8-12% SDS polyacrylamide gel electrophoresis (SDS PAGE; Mini Protean, Biorad, USA) and transferred onto nitrocellulose

membrane. The nitrocellulose membrane then was blocked with 5% bovine serum albumin (BSA) solution for 1 h and incubated with primary antibodies (4°C, overnight). Following that the membrane was washed three times and incubated with the appropriate horseradish peroxidase (HRP)-conjugated secondary antibody for 1 h. The membrane then was exposed to Immobilon Western Chemiluminescent HRP substrate (Merck Millipore) and the protein bands were visualized with chemiluminescence imaging system (Syngene, UK). Tris buffered saline with Tween 20 (TBST; 137 mM NaCl, 20 mM Tris-HCl pH 7.6, 0.05% Tween 20) was used for washing and preparing the blocking and antibody solutions. The expression of housekeeping protein, GAPDH, is used as protein loading control. The change in the protein expression level was semi-quantitatively determined with ImageJ. The following antibodies were utilized in the study: Anti-phospho VE-cadherin Y658 (Invitrogen, #441144G, dilution 1:1000), Anti-phospho VE-cadherin Y731 (Invitrogen, #441145G, dilution 1:1000); Anti-VE-cadherin (Clone D87F2, Cell Signaling Technology, #2500, dilution 1:1000); Anti-β-catenin (Clone H-102, Santa Cruz Biotechnology, #sc-7199, dilution 1:1000); Anti-GAPDH (Clone 14C10, Cell Signaling Technology, #2118, dilution 1:5000); HRP-conjugated anti-rabbit (Santa Cruz Biotechnology, #sc-2004, dilution 1:10000). The full scan of the immunoblots are provided in the Source Data file.

Viability assay measurement

The cells viability was ascertained following HMVECs exposure to Au NPs bearing different surface roughness and different size (1×10^9, 3×10^9, and 10×10^9 particles/mL). Briefly the HMVECs were grown in 96-wells clear bottom black plate and were exposed to the different AuNPs groups for 1, 12, and 24 h. Following the treatment, the dead cells were removed from the wells, and the adherent cells were washed once with phosphate buffered saline (PBS, pH 7.4). Afterward, the adherent cells were stained with 1 μg/mL of Hoescht 33342 (Life Technology, USA) DNA stain for 30 min. The stained cells then were washed once with PBS, prior reading the fluorescence emission of the Hoescht 33342 (Ex/Em: 350/461) with microplate reader (Hidex, Finland). Since only viable cells remain in the wells following the Au NPs exposure, the detected DNA content would proportionally correspond with the number of viable cells. The viability readout from the control group was used to normalize the samples' readout.

Membrane damage measurement

To determine the extent of membrane damage induced by the Au NPs bearing different surface roughness, HMVECs, which were grown in 96-wells clear bottom black plate, were exposed were exposed to the different groups of Au NPs (1×10^9, 3×10^9, and 10×10^9 particles/mL) for 1, 12, and 24 h. Thereafter, the exposed cells were washed once with PBS, and stained with of cell impermeant Nuclear blue DCS1 (10 µM; AAT Bioquest, USA) for 15 min. Thereafter, the cells were washed with PBS and double stained with cell permeant 1xSBYR Green I (Sigma Aldrich, USA) for another 15 min. The cells then were washed PBS, and the fluorescence signals were measured with Hidex microplate reader. Nuclear blue DCS1 signal was obtained at Excitation/Emission wavelengths of 350/461, while the SBYR Green I signal was obtained at Excitation/Emission wavelengths of 497/520. The number of dead cells were determined by normalizing the nuclear blue DCS1 l signal to the SBYR Green I signal. The dead cell number readout from the control group was used to normalize the samples' readout.

Reactive oxidative species (ROS) detection

Total intracellular ROS production was measured in the HMVECs, which were grown in 96-wells clear bottom black plate, following their exposure to 1×10^9, 3×10^9, and 10×10^9 particles/mL of Au NPs bearing different size and surface roughness. Following the Au NPs exposure (1, 6, and 12 h), the remaining Au NPs treatment suspension was removed, and the cells were washed once with PBS. Afterward, the cells were exposed to cocktail of oxidative stress indicator, CellROX Deep Red (5 µM, Life Technologies), and nucleus stain, Hoechst 33342 (1 µg/mL) for 30 min. Thereafter, the cells were washed twice with PBS. The intracellular ROS level was determined by normalizing the fluorescence signal of CellROX Deep Red with the signal derived from the Hoechst 33342 that indicates total cell number. The fluorescence readout obtained with Hidex microplate reader for excitation/emission wavelengths of 640/665 nm and 350/461 nm were used to determine the intensities of CellRox deep red and Hoechst 33342,

respectively. The intracellular ROS readout from the control group was used to normalize the samples' readout.

To determine the specific hydrogen peroxide (H_2O_2) production level, the exposed HMVECs (grown in 96-wells clear bottom black plate cells) were washed and exposed to cocktail of Cell Meter™ Intracellular Fluorimetric Hydrogen Peroxide Assay Kit with OxiVision™ Blue peroxide sensor (AAT Bioquest) and 1 x SBYR Green I nucleus stain. Following 30 min staining incubation, the cells were washed with PBS and the fluorescence signal was determined with Hidex microplate reader. The H_2O_2 level was determined by normalizing the fluorescence signal of OxiVision Blue with the signal derived from the SBYR Green I that indicates total cell number. The fluorescence readout obtained for excitation/emission wavelengths of 350/461 nm and 497/520 nm were used to determine the intensities of OxiVision Blue and SBYR Green I, respectively. The intracellular H_2O_2 level from the control group was used to normalize the samples' readout.

Au NPs deposition and internalization

Volumetric centrifugation method *in vitro* sedimentation, diffusion and dosimetry (VCM ISDD) protocol[28-30] was used to determine the Au NPs effective density by taking account their aggregate volume and their hydrodynamic size.

The estimated Au NPs deposited fraction (f_D) was fitted as Gompertz sigmodial function:

$$f_D(t) = 1 - e^{-\alpha t} \qquad \text{(Equation S1)}$$

Where α (h^{-1}) is the specific deposition fraction constant, and t (h) is the Au NPs exposure time.

The delivered Au NPs dose or Relevant *In vitro* Dose (RID_N) was estimated through the particle number of Au NPs deposition on the cell surface over time.

$$RID_N = (1 - e^{-\propto t}) \times N \qquad \text{(Equation S2)}$$

Where N is the total number of Au nanoparticles.

Internalization of the Au NPs was measured with inductively coupled plasma mass spectrophotometry (ICP-MS) Briefly, the HMVECs, which were grown in 6 cm dish, were exposed to Au NPs (6×10^9 particle/mL, 1 h) at 37ºC. Following the treatment, the cells then were washed, collected via trypsinization, and freeze dried. The dried cell pellet then was digested, and the resulting solution was filtered (pore size 0.45 μm) to remove any precipitates. Thereafter, the samples were analyzed for the Au content with ICP-MS (Agilent 7500, USA). The Au internalization was determined by normalizing the Au content to the dry cell weight (DCW).

Neovascularization detection in micrometastasis and NanoEL induction in micrometastasis

Female BALB/c mice (6-8 weeks) were inoculated with 4T1-EGFP cells (5×10^5 cells per mouse) *via* an injection into the mammary fat pad. Tumors were resected from the primary site when reaching ~500 mm^3. Immediately after surgery, the mice were intravenously administered with 10 mg/kg $Au_{30}R_3$. At 2.5 h after injection, the mice were intravenously injected with NPs-DiD (0.25 mg/kg). At 12 h post injection, PE-conjugated anti-CD31 antibody (Clone 390, Biolegend, #102408, 0.1 mg/kg) was intravenously injected to tag vessels. Then accumulation of NPs-DiD in lung metastases was observed using IVM imaging.

SUPPLEMENTARY FIGURES

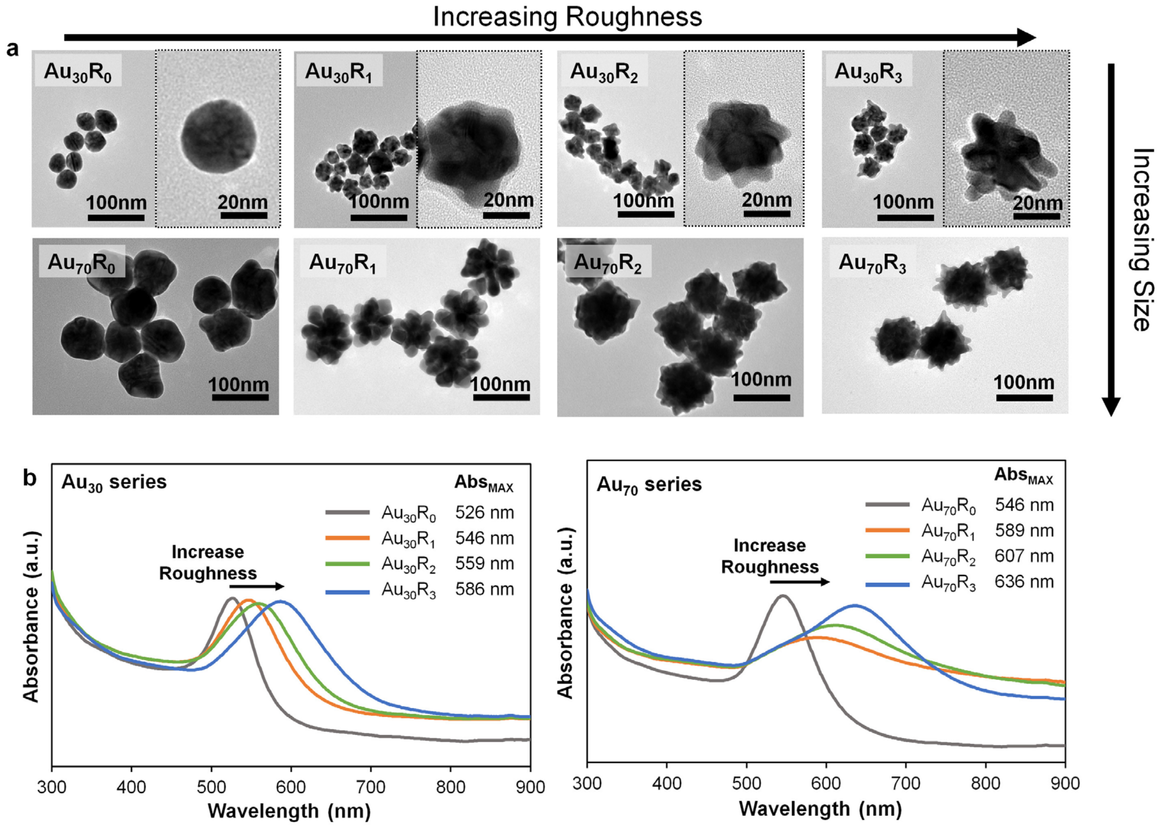

Supplementary Figure 1. Characterization of gold nanoparticles (Au NPs) with different surface roughness. (**a**) Transmission electron microscope (TEM) images depict the library of Au NPs with different primary sizes and different surface roughness. High magnification particle image demarcated with dotted line. The images shown are representative of three independent experiments. (**b**) UV-vis absorbance spectra of Au_{30} NPs series and Au_{70} NPs show significant red-shift with the increase of Au NPs surface roughness. Source data are provided as a Source Data file.

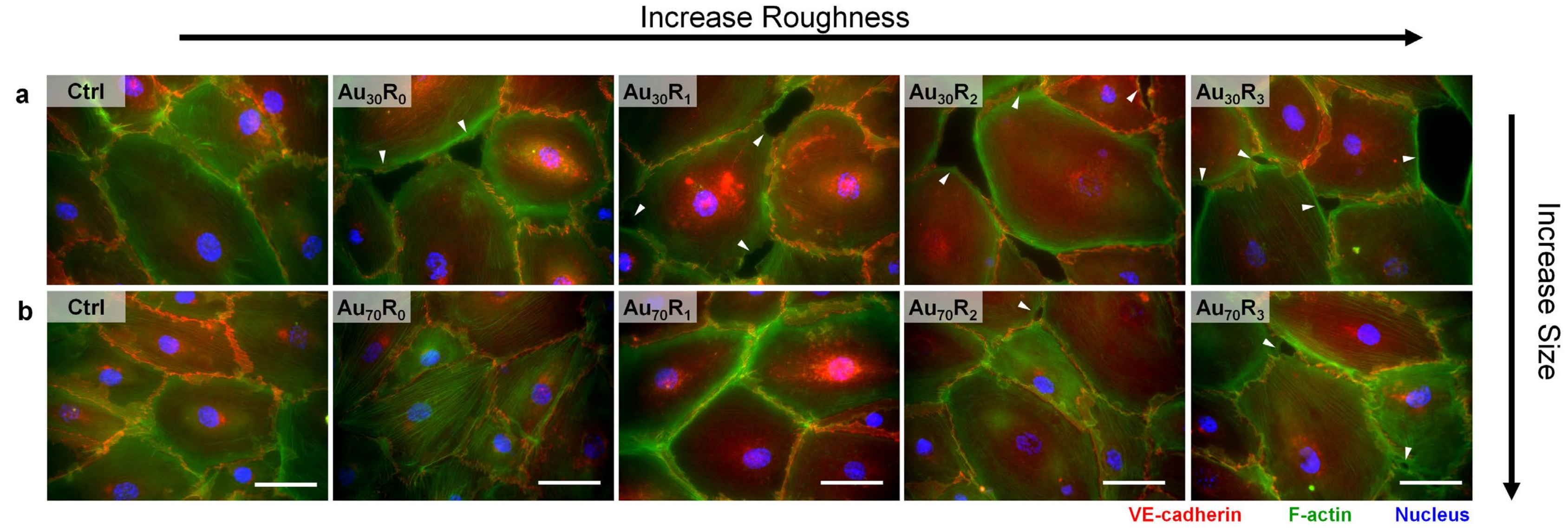

Supplementary Figure 2. Au NPs induced endothelial cell leakiness (Au NanoEL) in size and surface roughness dependent manner. Immunofluorescence images show the formation of intercellular gaps (white arrowheads) on the monolayer HMVECs barrier receiving 1 h treatment of 1×10^9 particle/mL of Au NPs the formed gap size was reduced with the increase of the Au NPs size. Interestingly, the size of the formed gaps was detected to increase proportionally with the increase of surface roughness in (**a**) Au_{30} NPs and (**b**) Au_{70} NPs series. Scale bar: 50 µm. Nucleus (blue), F-actin (green) and VE-cadherin (red). (**d**) The images shown are representative of three independent experiments.

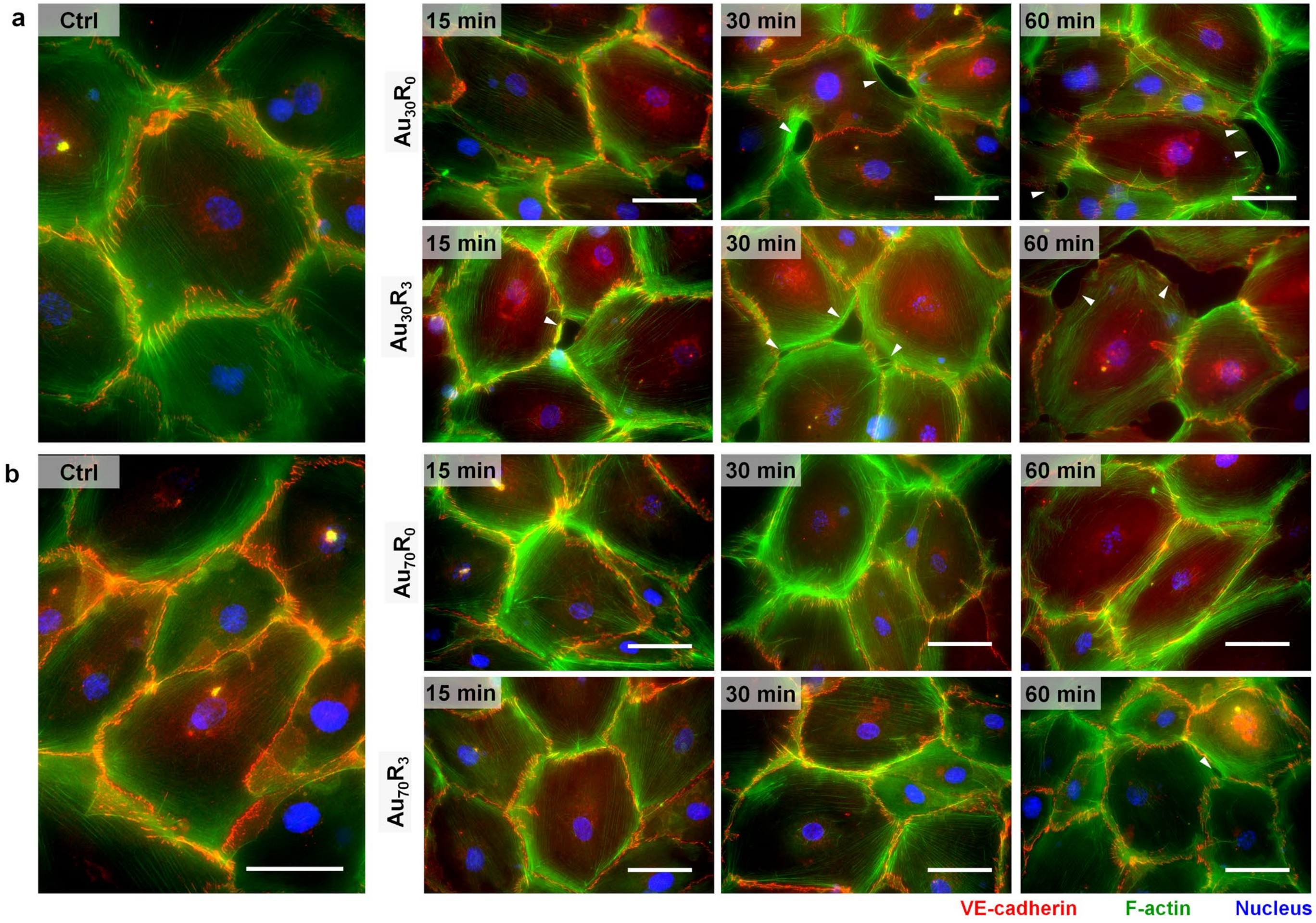

Supplementary Figure 3. Induction of Au NanoEL occurred in time dependent manner. Immunofluorescence images reveal the formation of intercellular gaps (white arrowheads) on the monolayer endothelial cell barrier receiving 1×10^9 particle/mL of (**a**) Au_{30} NPs and (**b**) Au_{70} NPs treatments. NanoEL was observed as early as 15 min exposure of $Au_{30}R_3$. Scale bar: 50 µm. Nucleus (blue), F-actin (green) and VE-cadherin (red). (**a, b**) The images shown are representative of three independent experiments.

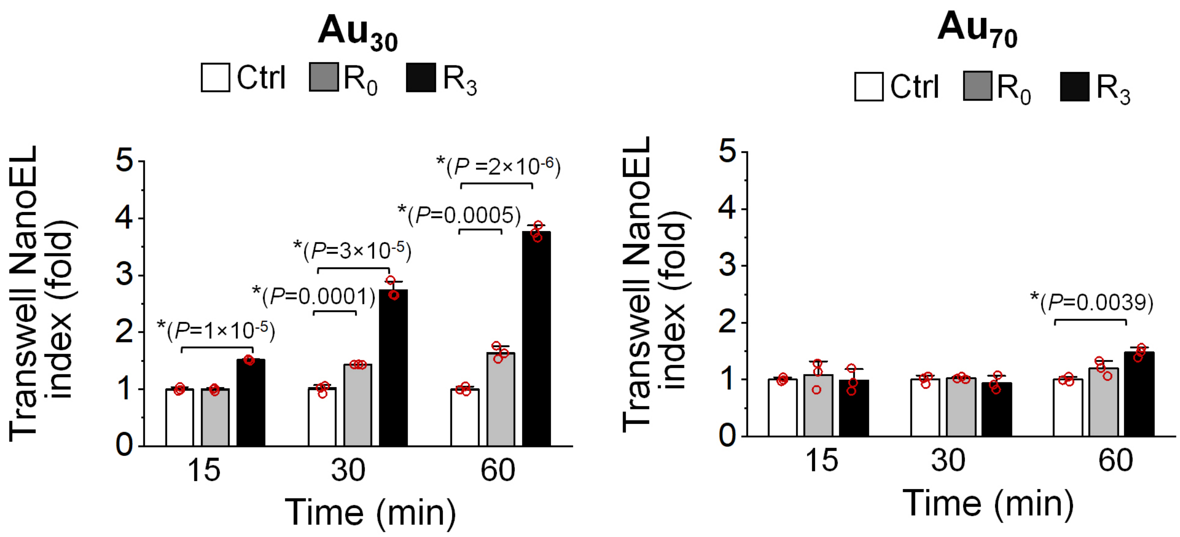

Supplementary Figure 4. Induction of Au NanoEL occurred in time dependent manner. NanoEL induction was detected to be time dependent. Data are average ± SD, n=3, Two-way repeated measures ANOVA Tukey HSD post-hoc test, *significant against control, P<0.05. Source data are provided as a Source Data file.

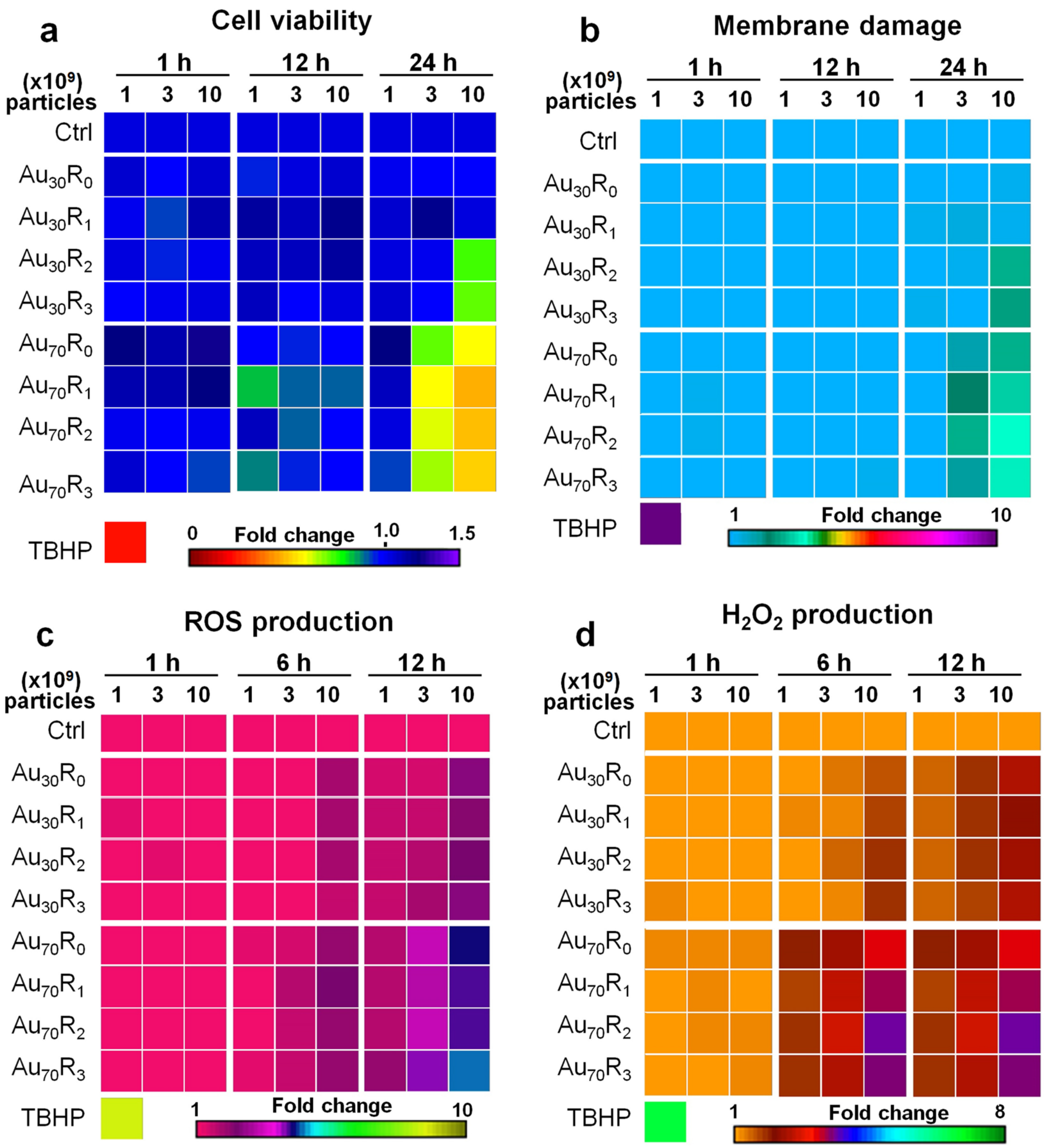

Supplementary Figure 5. Au NanoEL induction was independent of oxidative stress and cell death events. (**a**) Cell viability and (**b**) membrane damage profiles were unaffected significantly by short term Au NPs exposure (1 h), of which the initial NanoEL induction was observed. tert-Butyl hydro peroxide (TBHP; 200 µM, 6 h) was used as positive control. Data presented in the heat map are average of three biologically independent samples (n=3). (**c, d**) Cellular redox homeostasis remained largely unperturbed following short exposure (1 h) of Au NPs with significant increase in the intracellular reactive oxidative species (ROS) production after 6-12 h exposure of the cells with Au NPs. Heat map of (**c**) reactive oxygen species (ROS) and (**d**) hydrogen peroxide (H_2O_2) production in endothelial cells receiving Au NPs treatment (1 h). TBHP treatment (200 µM, 1 h) was used as positive control. Data presented in heat map are average of three biologically independent samples (n=3). Source data are provided as a Source Data file.

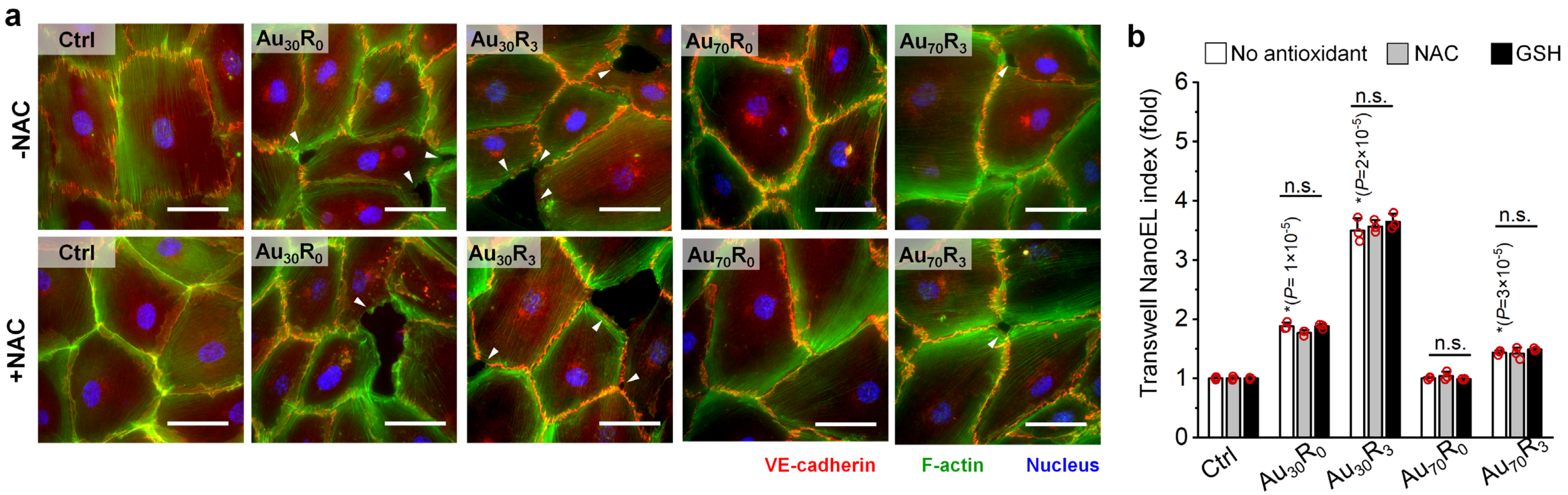

Supplementary Figure 6. Au NanoEL induction was independent of oxidative stress events. (**a**) Immunofluorescence images show anti-oxidant *N*-acetyl-cysteine (NAC,10 mM) did not alleviate the intercellular gaps formation on endothelial cell barrier exposed to Au NPs bearing the different surface roughness (1×10^9 particle/mL, 1 h). Scale bar: 50 µm. Nucleus (blue), F-actin (green) and VE-cadherin (red). The images shown are representative of three independent experiments. (**b**) NanoEL index on endothelial cell exposed to Au NPs with different size and surface roughness remain unchanged with anti-oxidant, NAC (10 mM) and L-glutathione (GSH; 10 mM) treatment. Data are mean ± SD, n=3, One-way ANOVA Tukey HSD post hoc test, *significant against control, P<0.05. Source data are provided as a Source Data file.

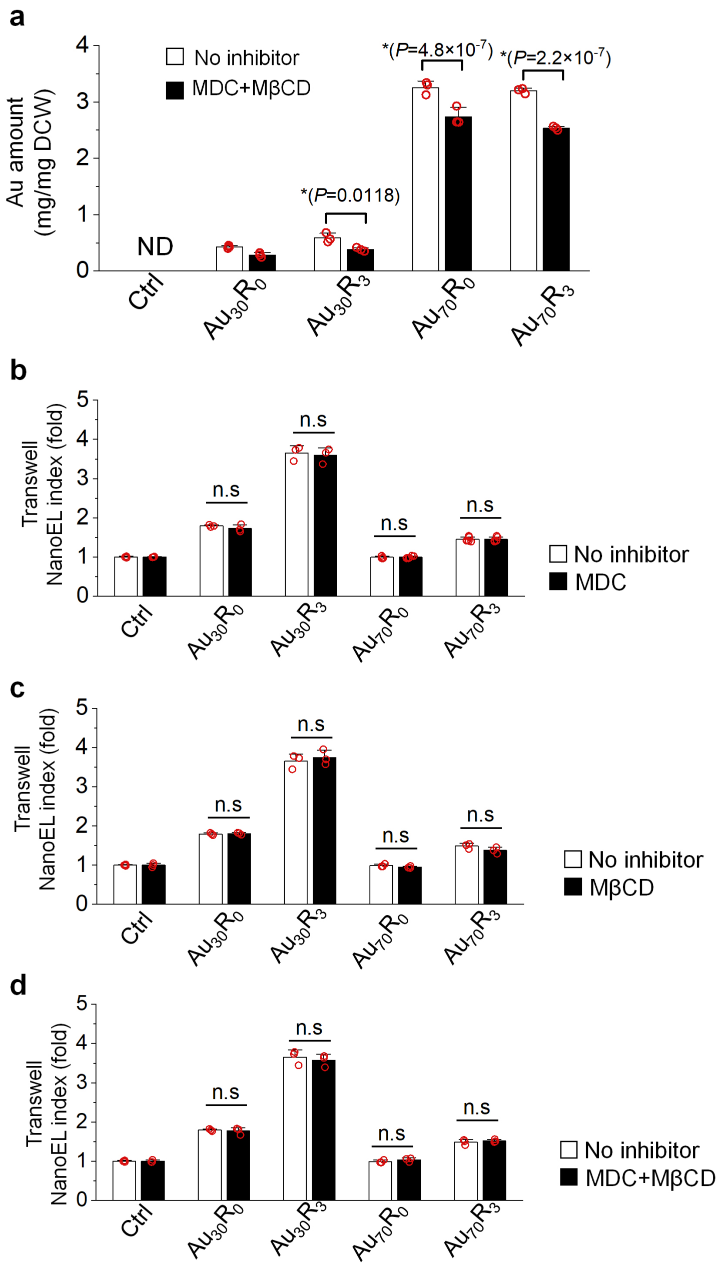

Supplementary Figure 7. Au NanoEL induction was independent of endocytosis process. (**a**) Increased presence of Au NPs on the endothelial cell monolayer to be dependent on the Au NPs size and not their surface roughness. Treatment of endocytosis inhibitors cocktail methyl-β-cyclodextrin (MβCD, 5 mM) and monodansycadaverine (MDC,10 µM), which blocks the caveolin and clathrin pathways, resulted in minimal change in the amount of Au detected through ICP MS analysis. Cells were exposed to Au NPs 6×10^9 particle/mL for 1 h. Data are average ± SD, n=3, One-way ANOVA Tukey HSD post-hoc test, *significant against compared group, $P<0.05$. Treating the endothelial monolayer with endocytosis inhibitors, (**b**) MDC (10 µM), (**c**) MβCD (5 mM), and (**d**) their cocktail (5 mM MβCD and 10 µM MDC), did not attenuate the NanoEL induction of these particles (1×10^9 particle/mL, 1h). Data are mean ± SD, n=3, One-way ANOVA Tukey HSD post-hoc test. Source data are provided as a Source Data file.

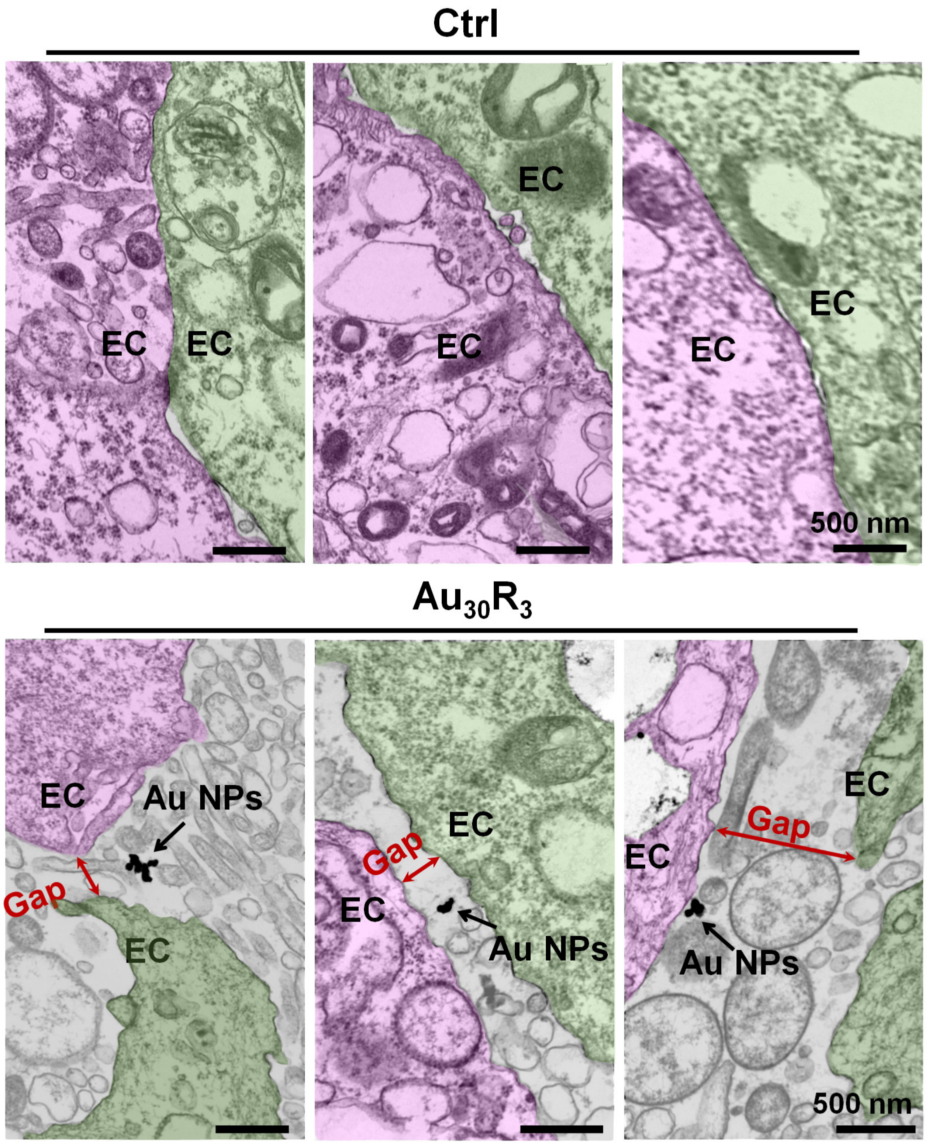

Supplementary Figure 8. NanoEL observed through transmission electron microscopy (TEM) imaging of ultrathin section of human umbilical vein endothelial cell (HUVEC) monolayers. NanoEL (Gap) was observed following 30 min exposure of $Au_{30}R_3$ in which the Au NPs were observed to be adjacent to the NanoEL site. In contrast, TEM imaging of the control ultrathin section showed intact adherens junction between two endothelial cells (ECs). Scale bar: 500 nm. Images from three independent experiments are shown.

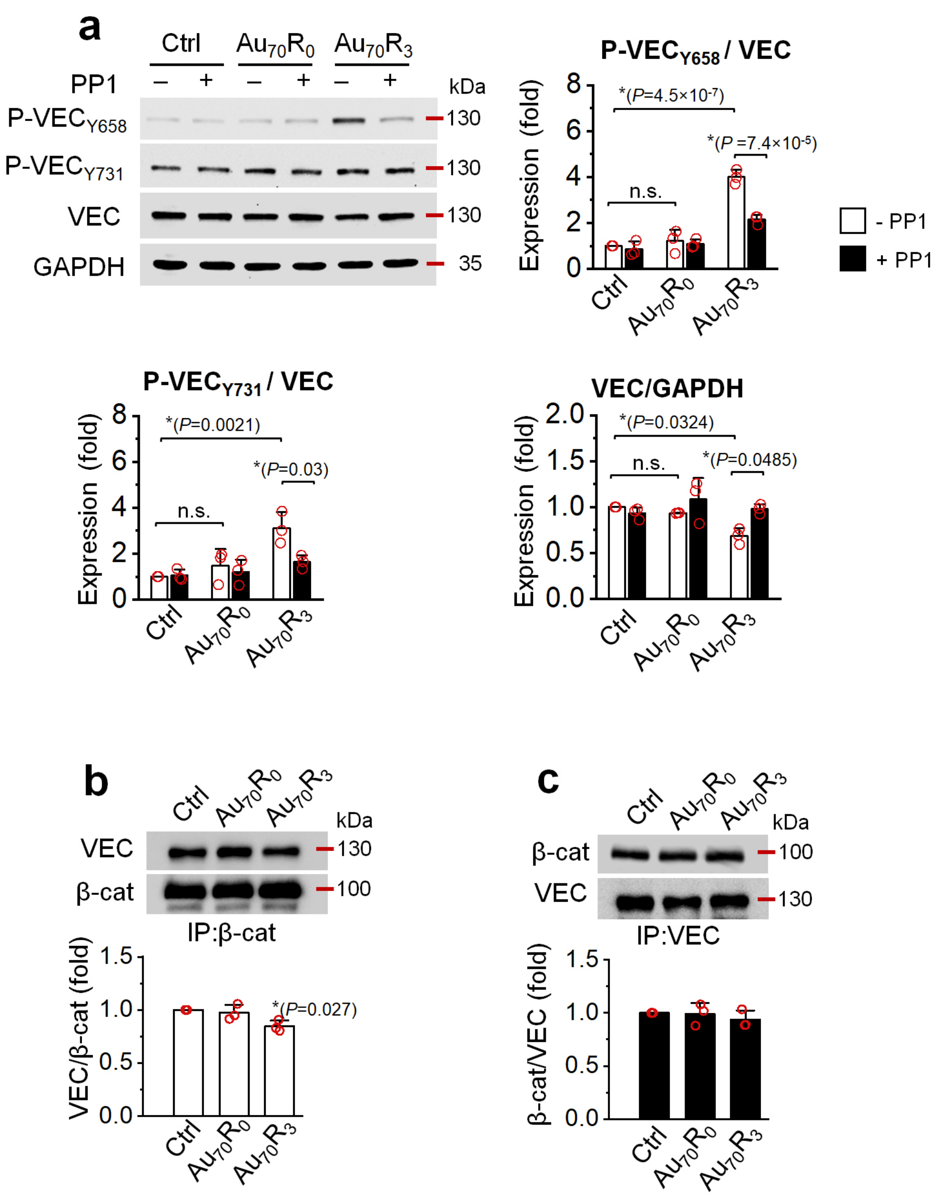

Supplementary Figure 9. (**a**) Immunoblotting (left panel) and its semi-quantitative analysis (right panel) show inhibiting VE-cadherin activation with Src kinase inhibitor, PP1 (10 µM) effectively repressed the VEC signaling activation. Inhibition was observed for the case of HMVECs treated (1h) with different surface roughness of Au_{70} NPs series at concentration of 1×10^9 particles/mL. Data are average ± SD, n=3, One-way ANOVA Tukey HSD post-hoc test, *significant against compared group, $P<0.05$. (**b, c**) Au NPs induced release of β-catenin from VE-cadherin. Protein samples of the same experiment were processed in parallel blots. (**b**) Immunoprecipitation of β-catenin (β-cat) shows reduced interaction between VEC and β-catenin (β-cat) detected following exposure to Au_{70} NPs (1×10^9 particles/mL, 1 h). (**c**) The vice versa immunoprecipitation of VE-cadherin (VEC) shows decreased amount of VE-cadherin (VEC) following exposure to Au_{70} NPs (1×10^9 particles/mL, 1 h). Data are mean ± SD, n=3, One-way ANOVA Tukey HSD post-hoc test, *significant against compared group, $P<0.05$, *, significant against control. Source data are provided as a Source Data file.

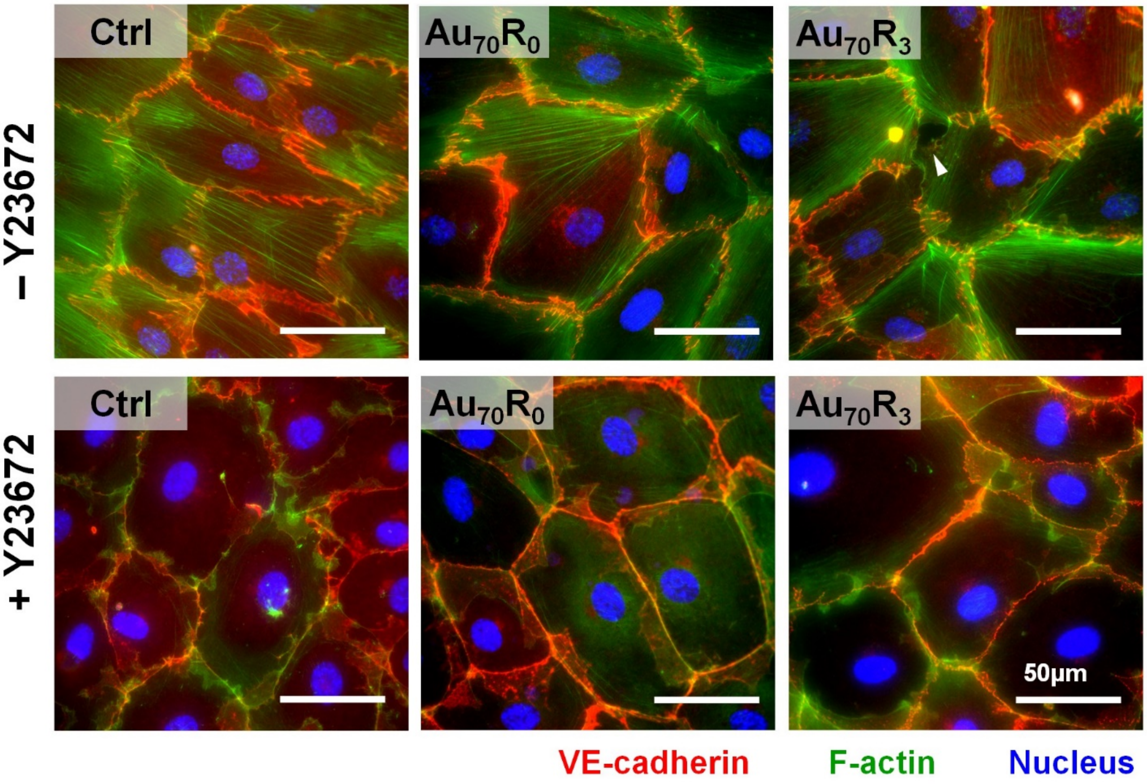

Supplementary Figure 10. Au NPs activated actin remodeling process that led to NanoEL induction. Negating the actin remodeling process with ROCK inhibitor, Y-27632 (10 μM, 1 h) abrogated the NanoEL induction. Immunofluorescence images depict significant reduction of the formed gap on monolayer endothelial cells following Y-27632 treatment. Scale bar: 50 μm. Nucleus (blue), F-actin (green) and VE-cadherin (red). The images shown are representative of three independent experiments.

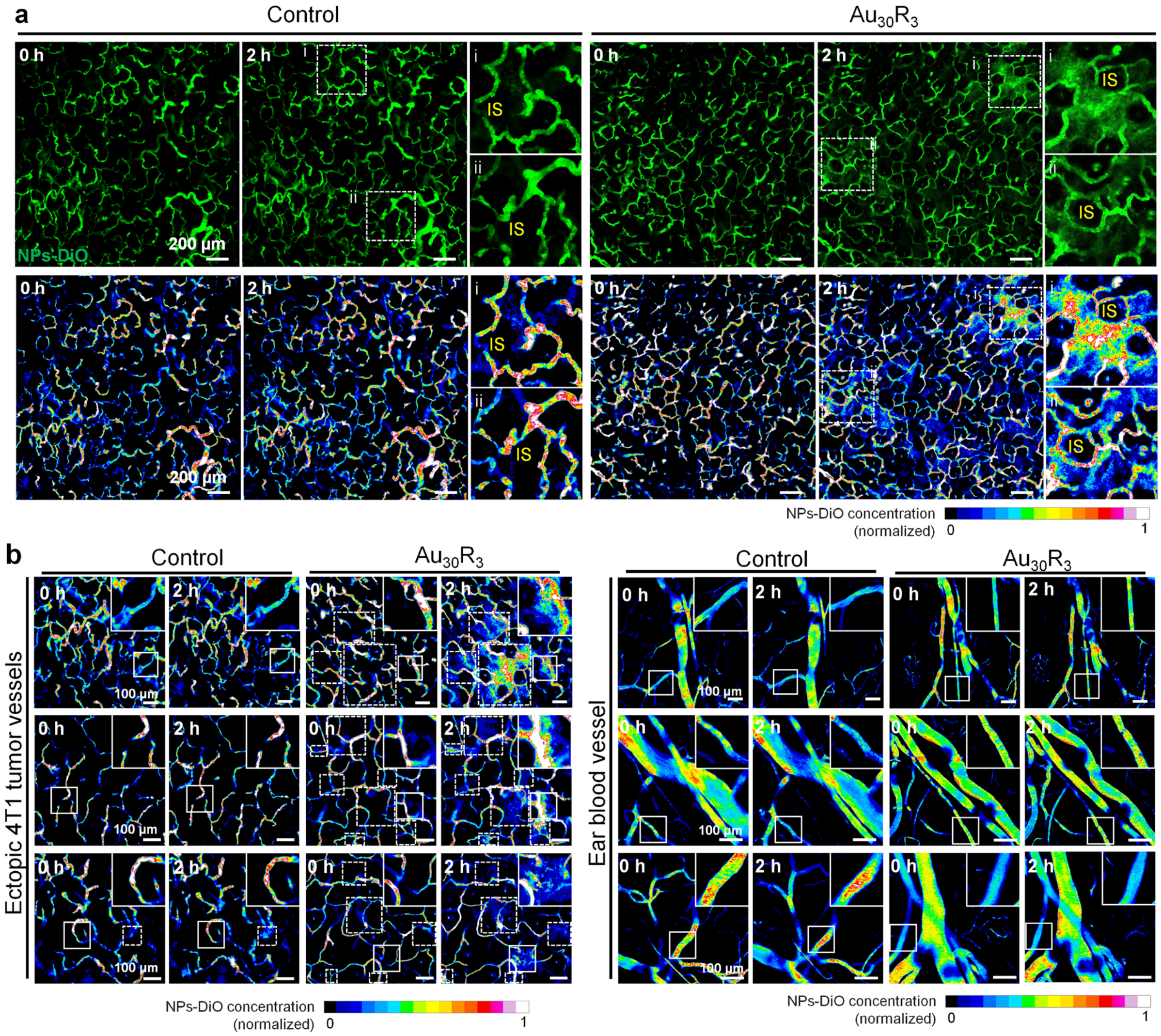

Supplementary Figure 11. NanoEL increased leakage of blood borne entities into the interstitial space of the ectopic 4T1 tumor. (**a**) Large area (~5.67 mm^2) fluorescent and multicolor view of the ectopic 4T1 tumor vasculature at the ear flap. Without NanoEL particles treatment, the tumor vasculature was marginally leaky (**i**,**ii**). However, with NanoEL treatment, leakiness greatly increased with obvious leakage of NPs-DiO from the blood vessels to the interstitial space (IS) (**i**,**ii**). Scale bar: 200 μm. The images shown are representative of three independent experiments. (**b**) Representative multicolor view of the ear flap from each mouse in study (n = 3 mice/group). Consistently leakiness into the interstitial space in the 4T1 ectopic tumor vasculature in the $Au_{30}R_3$ nanoparticles group, whereas no observable leakiness was observed at the other ear blood vessels of the same animal despite subjected to the same tail vein intravenous introduction of $Au_{30}R_3$. Scale bar: 100 μm.

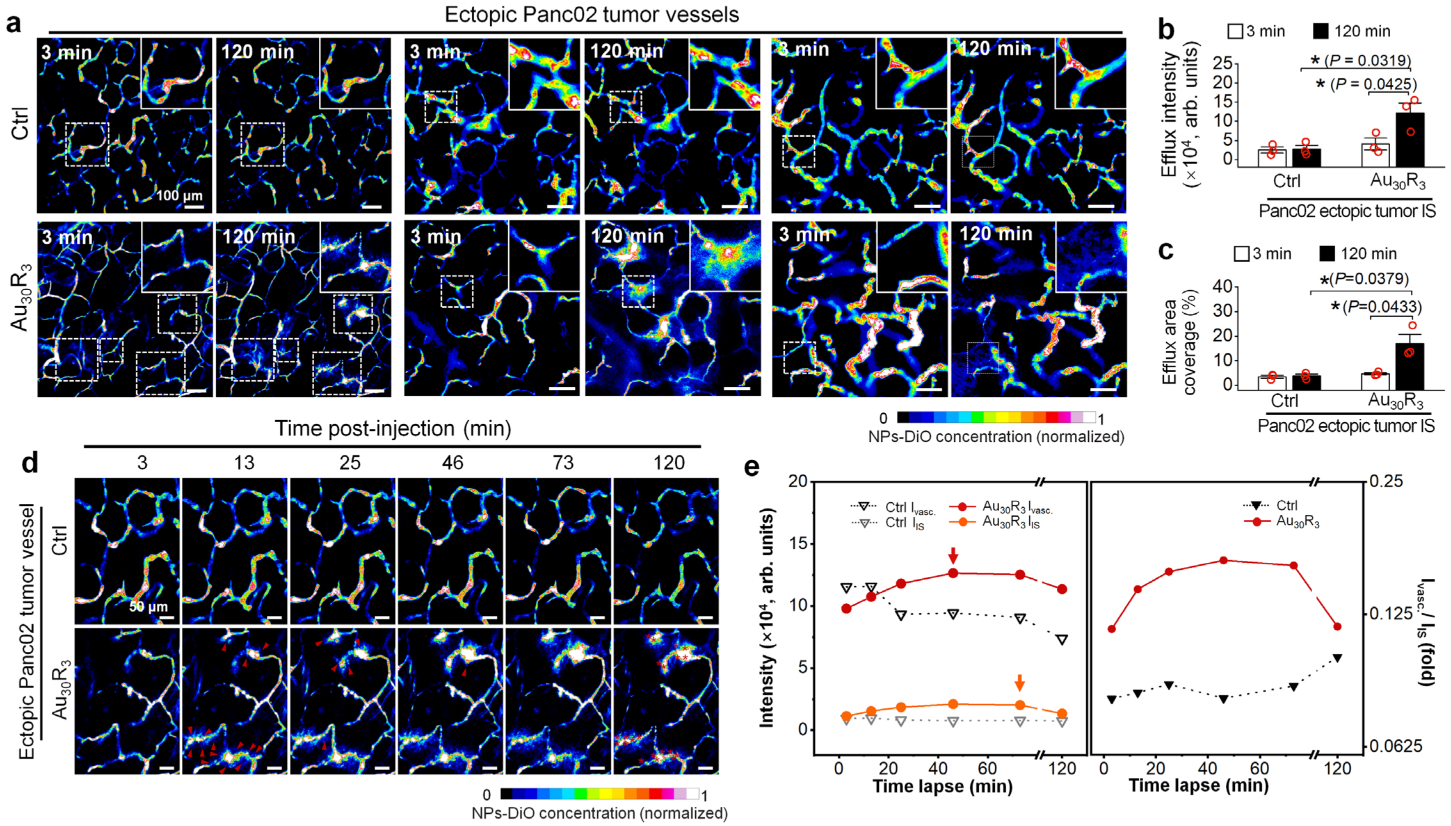

Supplementary Figure S12. NanoEL increased leakage of blood borne entities into the interstitial space of the ectopic Panc02 tumor. Murine Panc02 pancreatic tumor cells were ectopically implanted on the mouse ear flap (n=3 mice/group). Thereafter, the mice were treated either with vehicle control or NanoEL particles, $Au_{30}R_3$ (10 mg/kg). NPs-DiO (~100 nm) were used as common visualization particles, and the *in vivo* leakiness was viewed through intravital imaging (IVM). (**a**) Multicolor images show increased leakiness into the interstitial space in Panc02 ectopic tumor vessels in the $Au_{30}R_3$ nanoparticles group. Scale bar: 100 µm. Quantification of (**b**) the efflux intensity and (**c**) area of the interstitial area showed that $Au_{30}R_3$ has caused profound leakiness. Data are mean ± SEM, n= 3 mice/group, Two-way repeated measures ANOVA Tukey post-hoc test, *significant against compared group, $P<0.05$. (**d, e**) Evolution of leakiness of a focused area in CT26 ectopic tumor vasculatures. Profiling of NPs-DiO particles load in the vasculature versus interstitial space. The load peaks earlier in the vasculature (red arrow) than in the interstitial space (orange arrow). Scale bar: 50 µm. (**d**) The images shown are representative of three independent experiments. Source data are provided as a Source Data file.

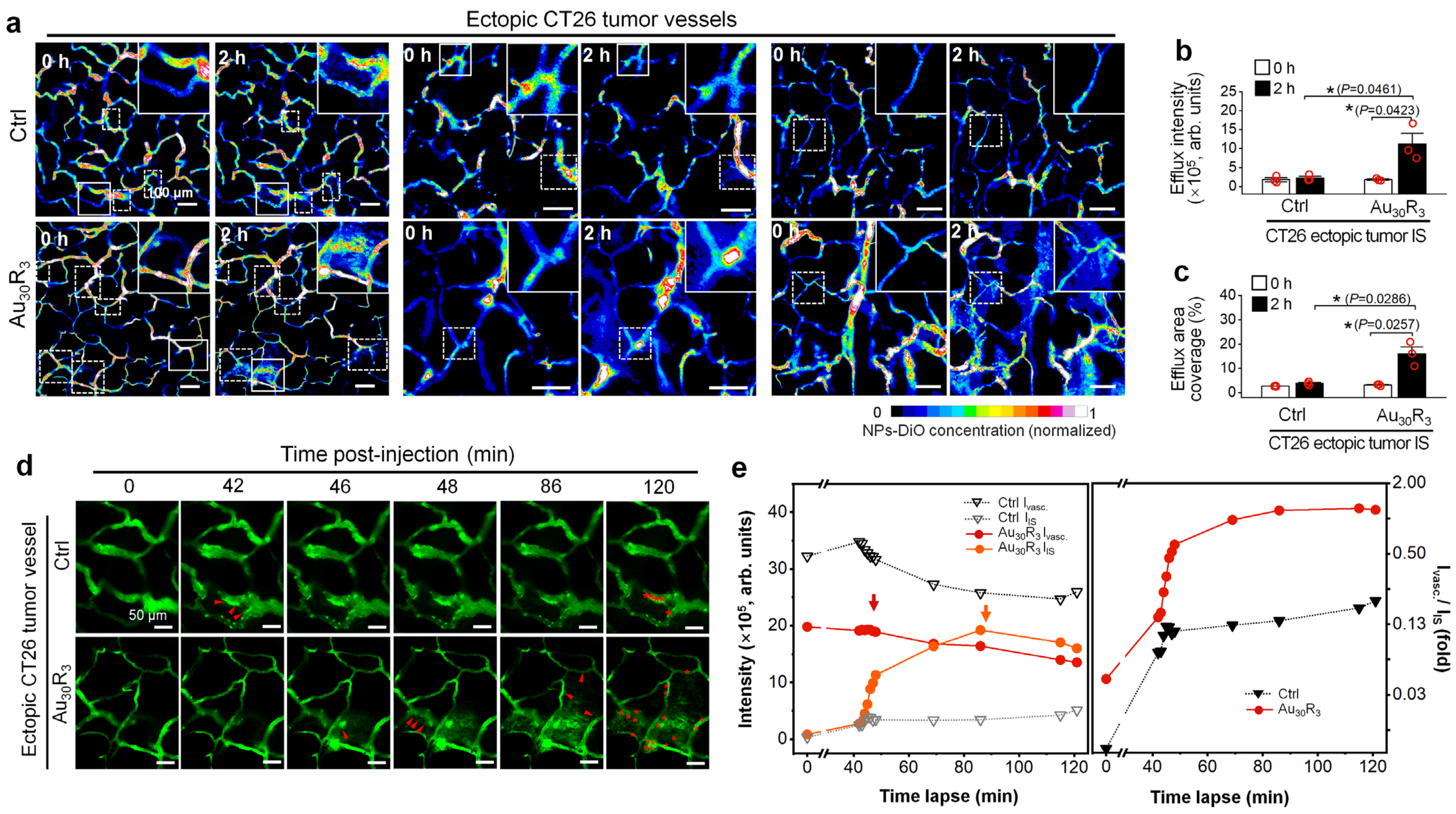

Supplementary Figure 13. NanoEL increased leakage of blood borne entities into the interstitial space of the ectopic CT26 hyperpermeable tumor. Murine CT26 hyperpermeable colorectal tumor cells were ectopically implanted on the mouse ear flap (n=3 mice/group). Thereafter, the mice were treated either with vehicle control or NanoEL inducing particles, $Au_{30}R_3$ (10 mg/kg). NPs-DiO (~100 nm) were used as common visualization particles, and the *in vivo* leakiness was viewed through intravital imaging (IVM). (**a**) Multicolor images show leakiness into the interstitial space in CT26 ectopic tumor vessels in both in control and the $Au_{30}R_3$ nanoparticles group. Scale bar: 100 µm. Quantification of (**b**) the efflux intensity and (**c**) area of the interstitial area showed significant change in the leakiness following $Au_{30}R_3$ treatment. Data are mean ± SEM, n=3 mice/group, Two-way repeated measures ANOVA Tukey post-hoc test, *significant against compared group, $P<0.05$. (**d, e**) Evolution of leakiness of a focused area in CT26 ectopic tumor vasculatures. Profiling of NPs-DiO particles load in the vasculature versus interstitial space. The load peaks earlier in the vasculature (red arrow) than in the interstitial space (orange arrow). Scale bar: 50 µm. (**d**) The images shown are representative of three independent experiments. Source data are provided as a Source Data file.

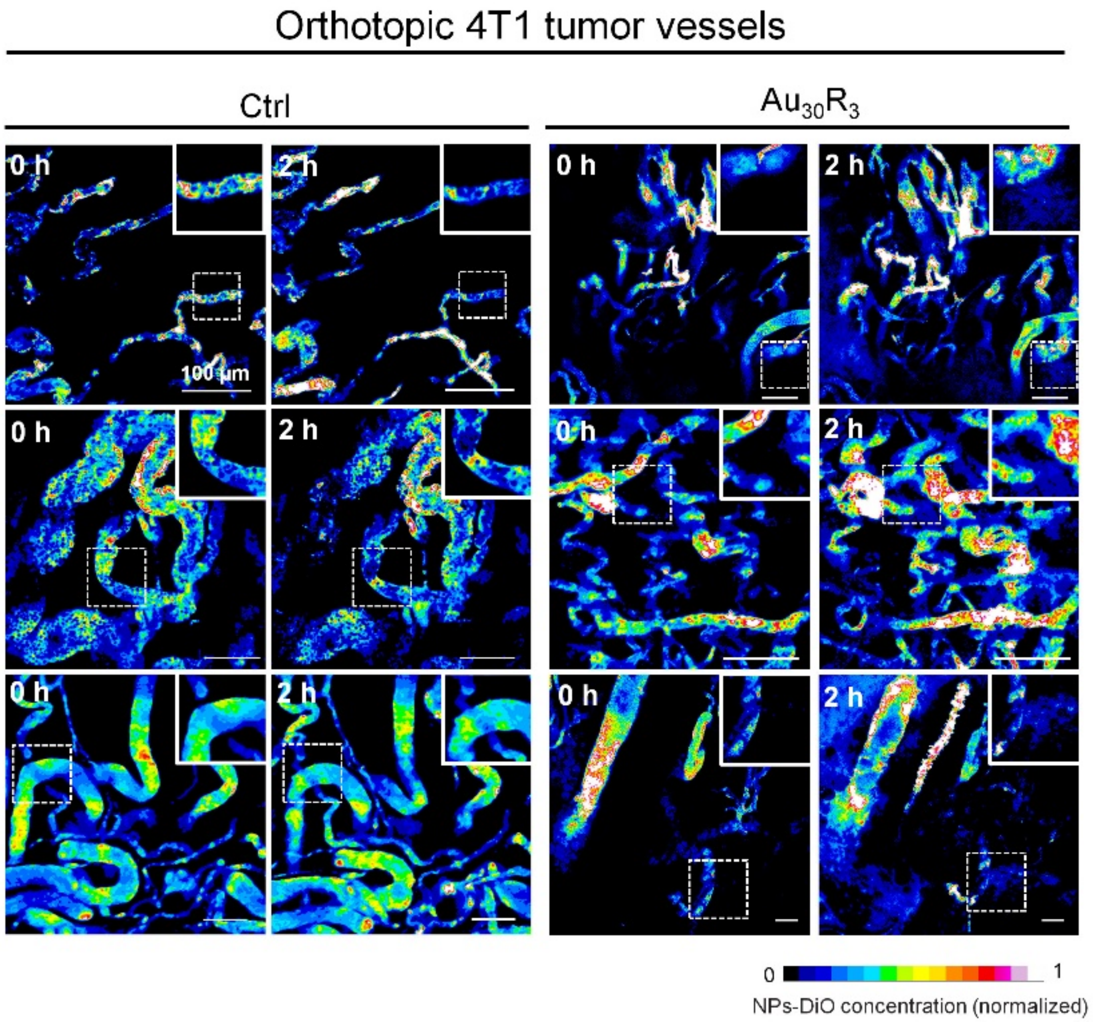

Supplementary Figure 14. NanoEL increased leakage of blood borne entities into the interstitial space of the orthotopic 4T1 tumor. Representative multicolor view of the orthotopic 4T1 tumor vasculature from each mouse in study (n = 3 mice /group). Without NanoEL particles treatment, the tumor vasculature was marginally leaky. However, with NanoEL treatment, leakiness greatly increased with obvious leakage of NPs-DiO from the blood vessels to the interstitial space (IS). Scale bar: 100 µm.

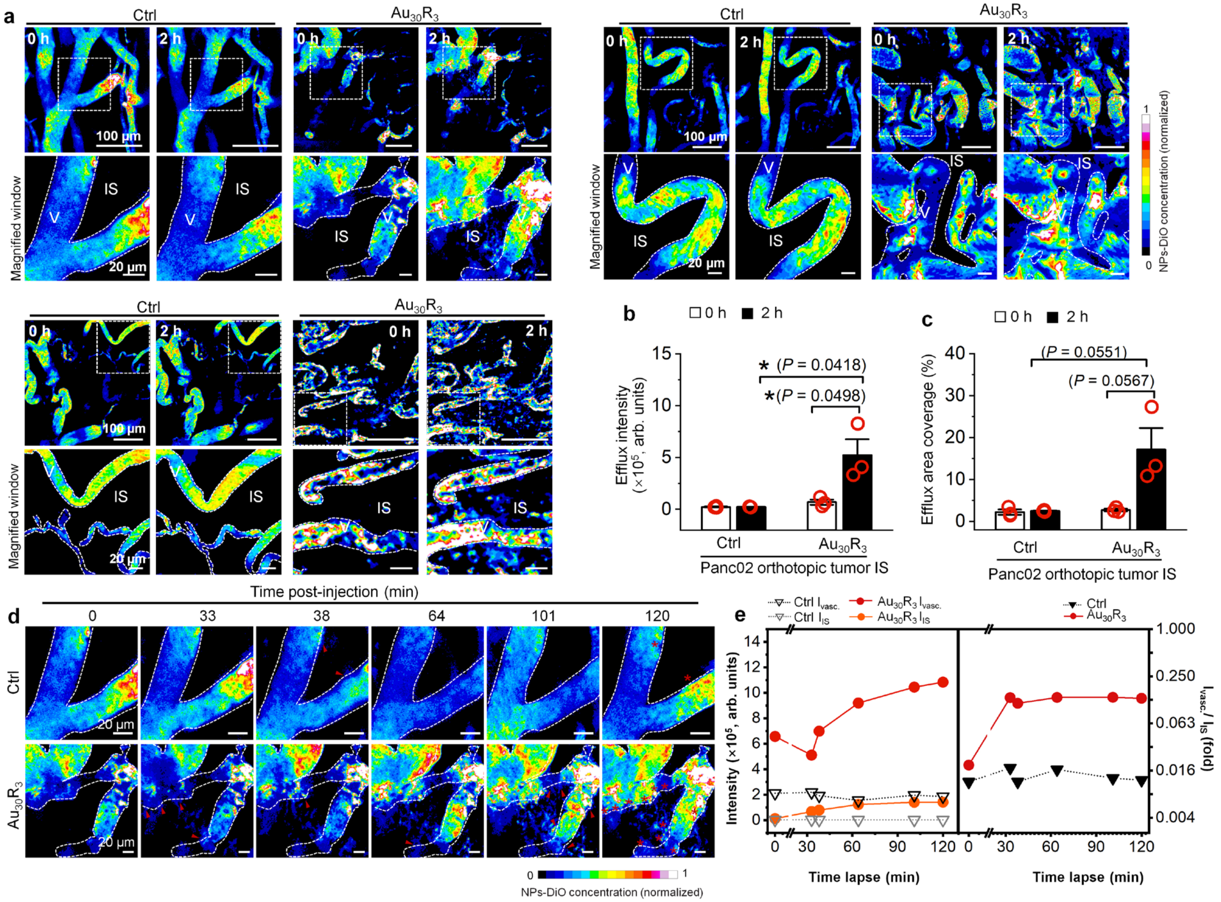

Supplementary Figure 15. NanoEL increased leakage of blood borne entities into the interstitial space of the orthotopic Panc02 tumor. Murine Panc02 pancreatic tumor cells were orthotopically implanted on the mice (n=3 mice/group). Thereafter, the mice were treated either with vehicle control or NanoEL particles, $Au_{30}R_3$ (10 mg/kg). NPs-DiO (~100 nm) were used as common visualization particles, and the *in vivo* leakiness was viewed through intravital imaging (IVM). (**a**) Multicolor images from each mouse in study show increased leakiness into the interstitial space in Panc02 orthotopic tumor vessels in the $Au_{30}R_3$ nanoparticles group. Scale bar: 100 µm. Quantification of (**b**) the efflux intensity and (**c**) area of the interstitial area showed that $Au_{30}R_3$ has caused profound leakiness. Data are mean ± SEM, n= 3 mice/group, Two-way repeated measures ANOVA Tukey post-hoc test, *significant against compared group, $P<0.05$. (**d, e**) Evolution of leakiness of a focused area in Panc02 orthotopic tumor vasculatures. Scale bar: 20 µm. (**d**) The images shown are representative of three independent experiments. Source data are provided as a Source Data file.

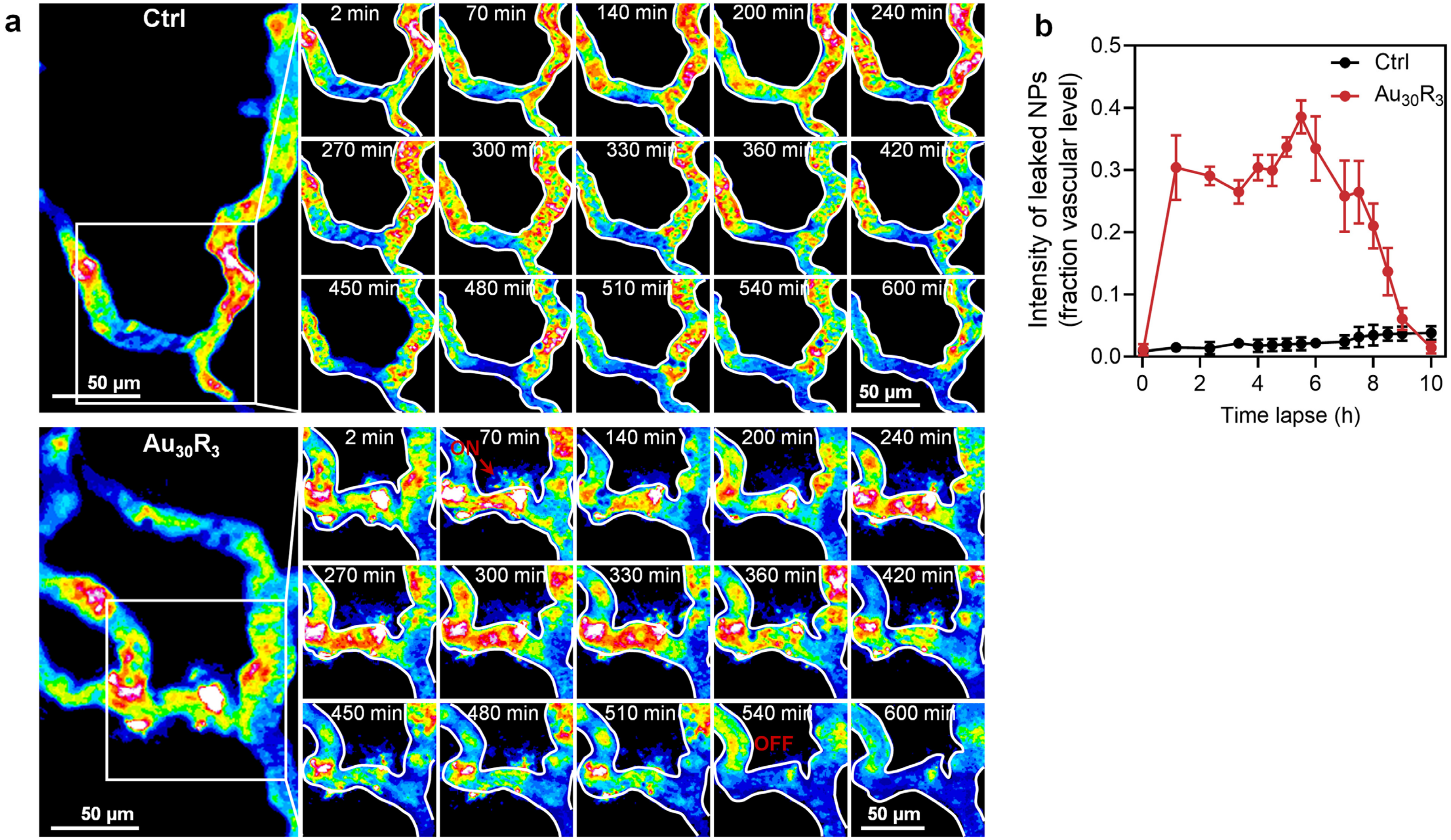

Supplementary Figure 16. Transient induction of NanoEL in 4T1 tumor model. Murine 4T1 breast tumor cells were ectopically implanted on the mice (n=3 mice/group). Thereafter, the mice were treated either with vehicle control or NanoEL particles, $Au_{30}R_3$ (10 mg/kg). NPs-DiO (~100 nm) were used as common visualization particles, and the *in vivo* leakiness was viewed through intravital imaging (IVM) over the course of 10 hours. (**a**) Representative multicolor images taken over the course of 10 hours show increased leakiness in $Au_{30}R_3$ nanoparticles group is transient in nature. Scale bar: 50 µm. (**b**) Corresponding quantification of the efflux intensity in the interstitial area gave evidence on the temporal effect of $Au_{30}R_3$ induced leakiness. Data are mean ± SEM, n= 3 mice/group.

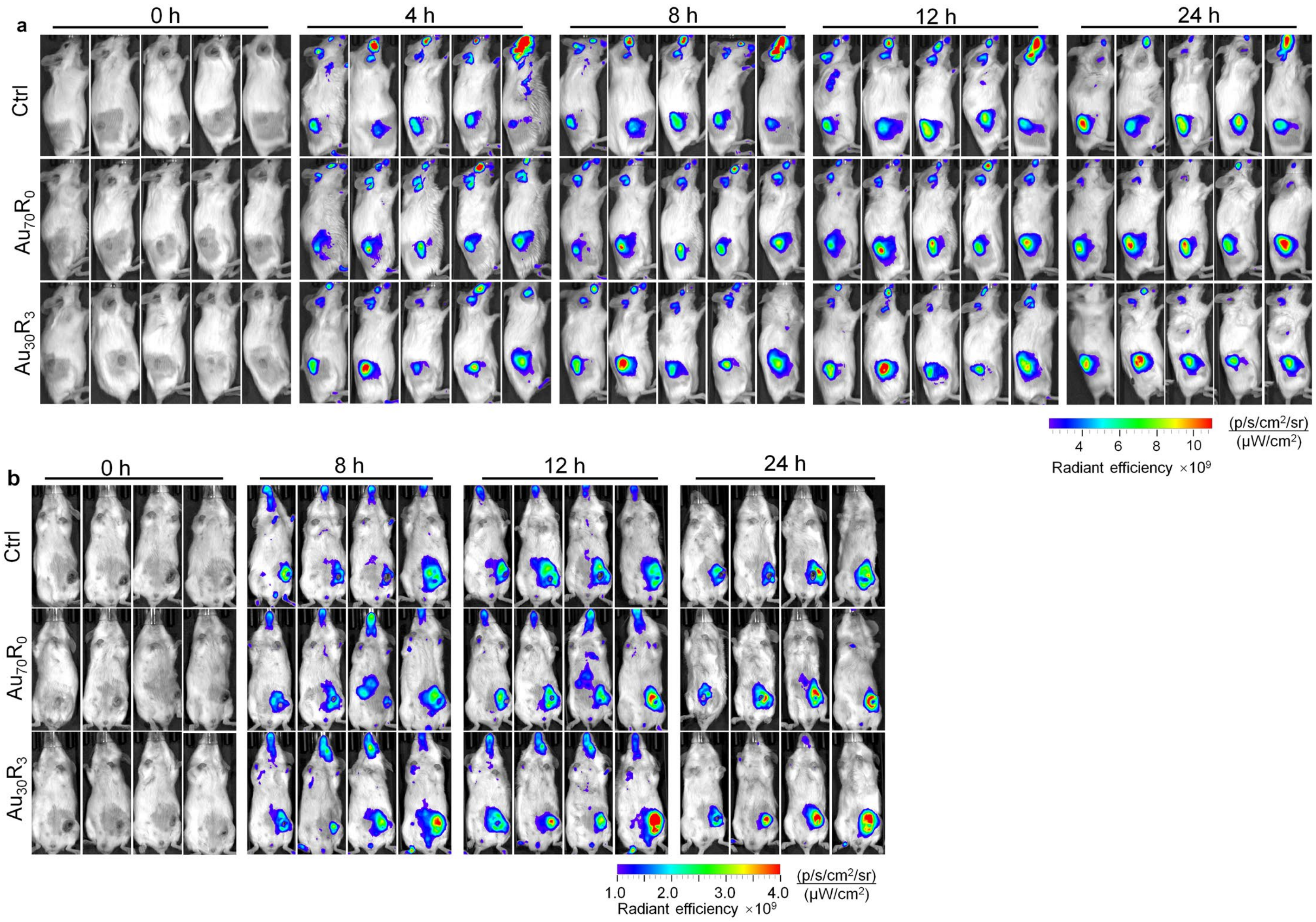

Supplementary Figure 17. NanoEL particles increases access to tumor with high penetration. Live animal imaging on (**a**) ectopic (n=5 mice/group) and (**b**) orthotopic (n=4 mice/group) 4T1 tumors over time course up to 24 hours. Faster and higher accumulation of fluorescent particles at the tumor in the NanoEL particles ($Au_{30}R_3$ and $Au_{70}R_0$) as compared to the vehicle-treated control. In the $Au_{30}R_3$ group, saturated levels were quickly achieved and persists throughout the duration of the entire study.

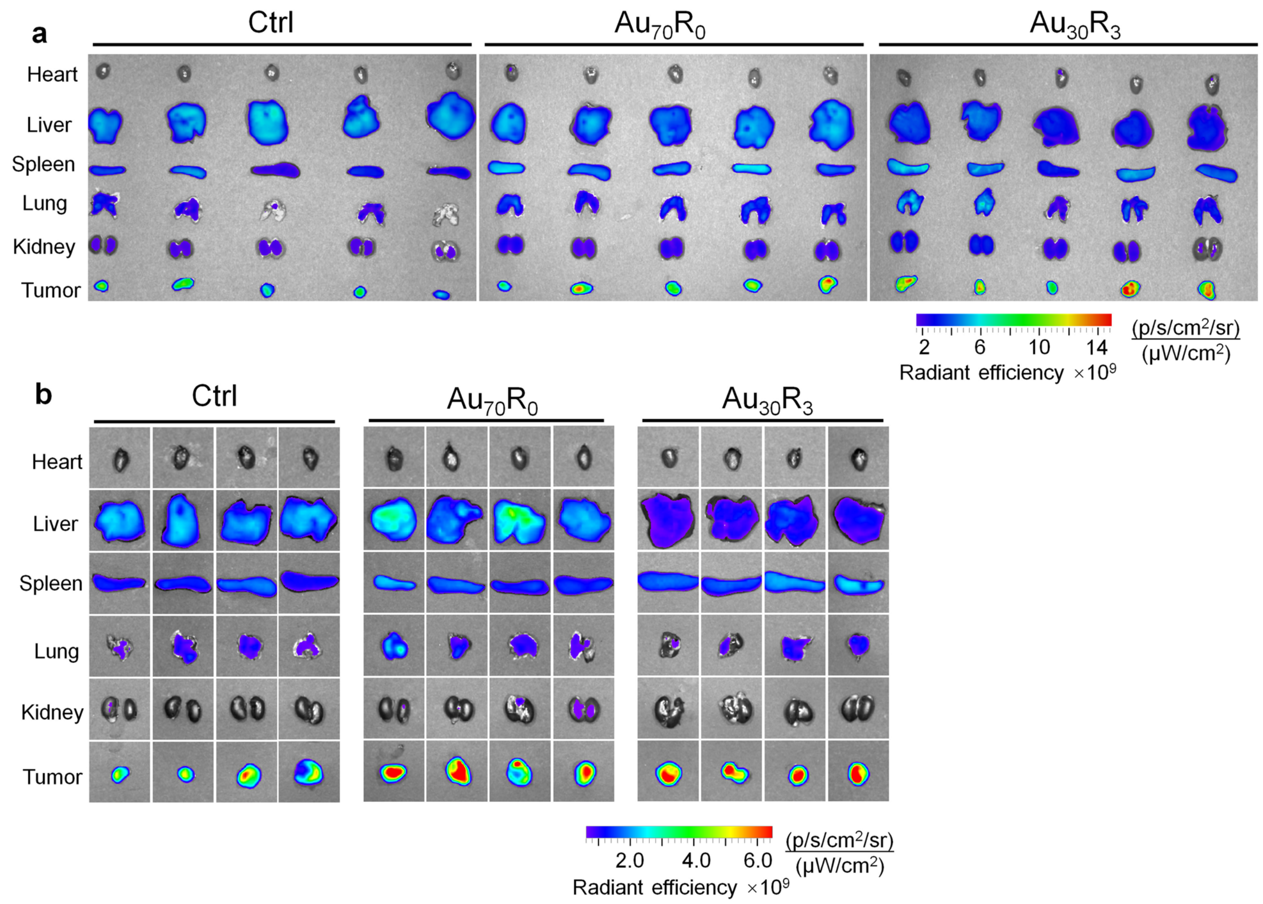

Supplementary Figure 18. NanoEL particles increases access to tumor with high penetration. Various organs harvested from the mice with (**a**) ectopic (n=5 mice/group) and (**b**) orthotopic (n=4 mice/group) 4T1 tumors receiving $Au_{30}R_3$ and $Au_{70}R_0$ NPs treatment were scanned with IVIS imaging system to visualize the accumulation of tracer particles, 100 nm NPs-DiD. In the highly NanoEL inducing particle group, $Au_{30}R_3$ showed the highest accumulation of fluorescent particles in the tumor yet with the lowest accumulation in the liver, an otherwise common site of nanoparticles accumulation.

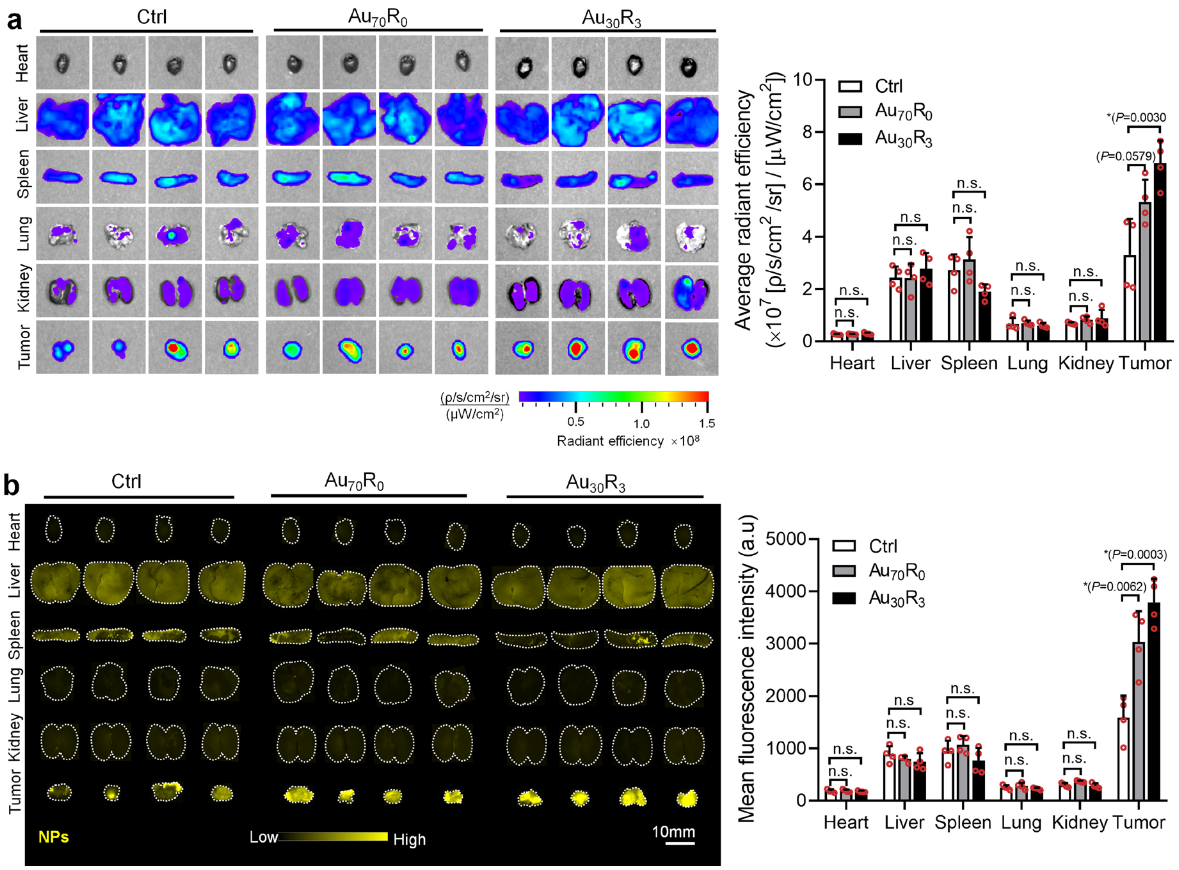

Supplementary Figure 19. NanoEL particles increases vascular leakiness resulting in deeper tumoral penetration in pancreatic tumor model. Murine Panc02 pancreatic tumor cells were orthotopically implanted on the mouse (n=4 mice/group). Thereafter, the mice were treated either with vehicle control, or NanoEL inducing particles, $Au_{30}R_3$ and $Au_{70}R_0$. Common visualization particles NPs-RhoB (~110 nm) were utilized. After 24 hours, the animals were sacrificed, and their various organs were scanned for fluorescence with IVIS and fluorescence reflectance imaging. Both (**a**) IVIS imaging and (**b**) fluorescence reflectance imaging show highest accumulation of highly NanoEL inducing particle group, $Au_{30}R_3$ in the tumor. Data are mean ± SEM, One-way ANOVA Tukey HSD post-hoc test, *significant against control, $P<0.05$. Source data are provided as a Source Data file.

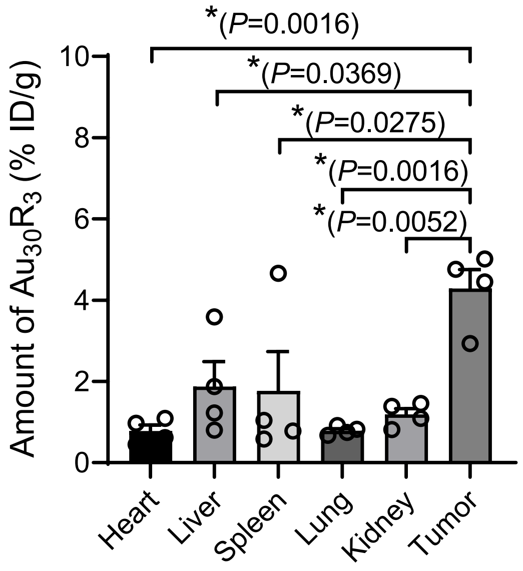

Supplementary Figure 20. Au NPs distributions in major organs and tumors. Increased Au NPs accumulation was observed in tumors. Murine breast cells 4T1 (5×10^5 cells) were ectopically implanted on female BALB/c mice (n = 4/group). Mice were intravenously injected with $Au_{30}R_3$ NPs (10 mg/kg, containing 1% BSA). At 12 h after injection, the organs of mice including heart, liver, spleen, lung, kidney, and tumor were harvested, rinsed with PBS, weighed after removing excess fluid and homogenized. Then the accumulated Au NPs in the organs were quantified using inductively coupled plasma mass spectroscopy (ICP-MS). Data are mean ± SEM, One-way ANOVA Tukey HSD post-hoc test, *significant against compared groups, $P<0.05$. Source data are provided as a Source Data file.

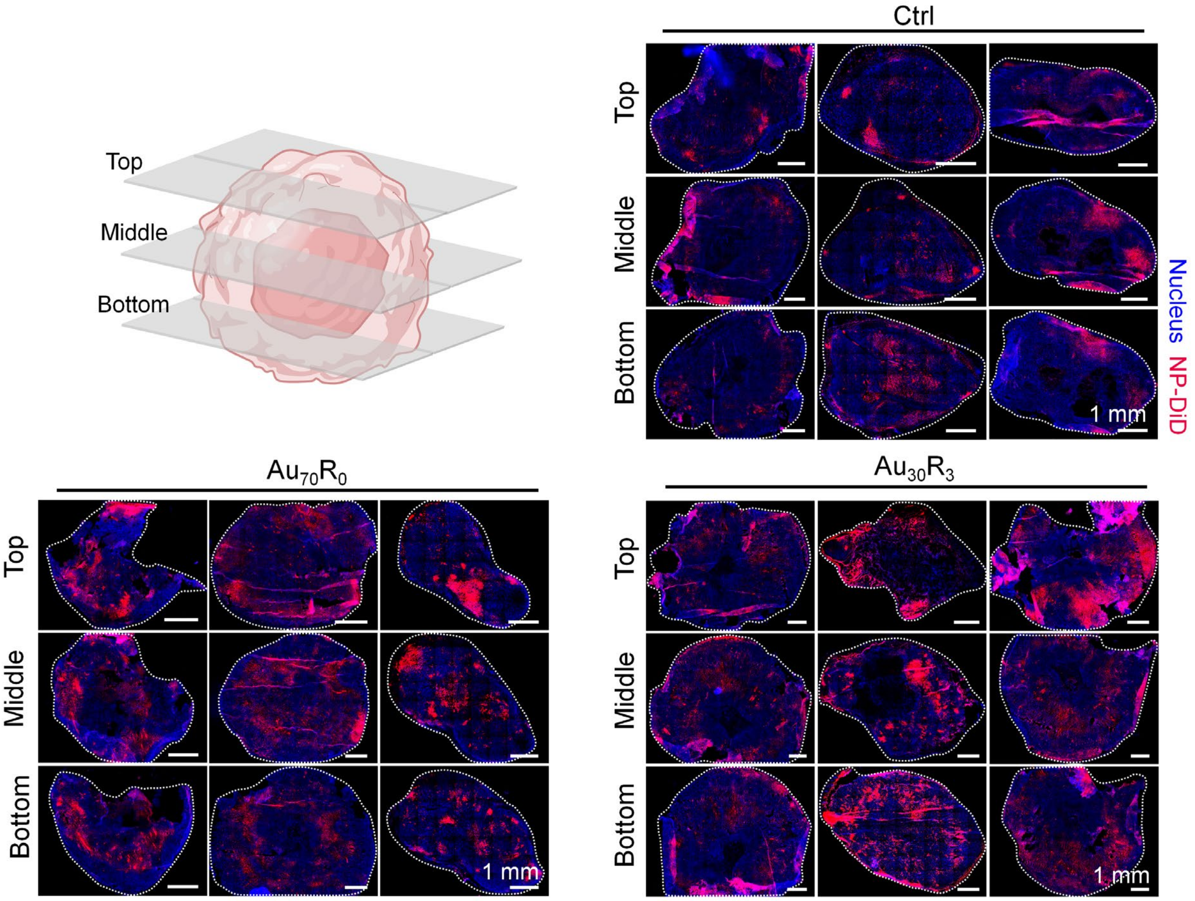

Supplementary Figure 21. NanoEL particles increases vascular leakiness resulting in deeper tumoral penetration in 4T1 ectopic tumor model. 4T1 ectopic tumor sections revealed that control group showed little penetration of the NPs-DiD into the interior sections of the tumor while $Au_{70}R_0$ and $Au_{30}R_3$ visually showed significantly higher penetration of NPs-DiD into the 4T1 ectopic tumor. Scale bar: 1 mm.

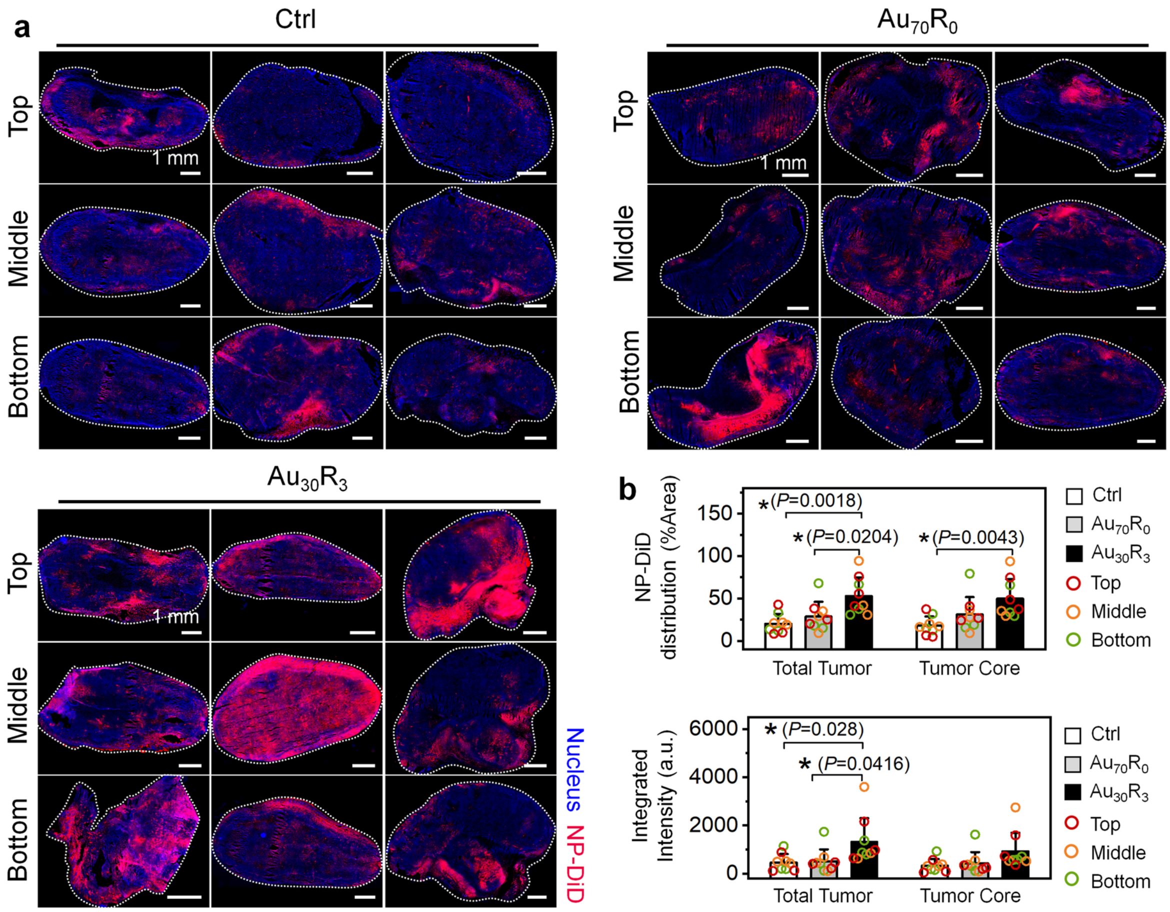

Supplementary Figure 22. NanoEL particles increases vascular leakiness resulting in deeper tumoral penetration in 4T1 orthotopic tumor model. (**a**) 4T1 orthotopic tumor sections revealed that control group showed little penetration of the NPs-DiD into the interior sections of the tumor while $Au_{70}R_0$ and $Au_{30}R_3$ visually showed significantly higher penetration of NPs-DiD into the tumor. Scale bar: 1 mm. (**b**) Quantification of the NPs-DiD coverage area and intensity in the representative images of 4T1 orthotopic tumor sections showed deeper and higher concentration of NPs-DiD penetration into the core regions of the tumor. Data are mean ± SD, n=9 independent tumor sections, N=3 mice/group, One-way ANOVA Tukey HSD post-hoc test, *significant against control, $P<0.05$. Source data are provided as a Source Data file.

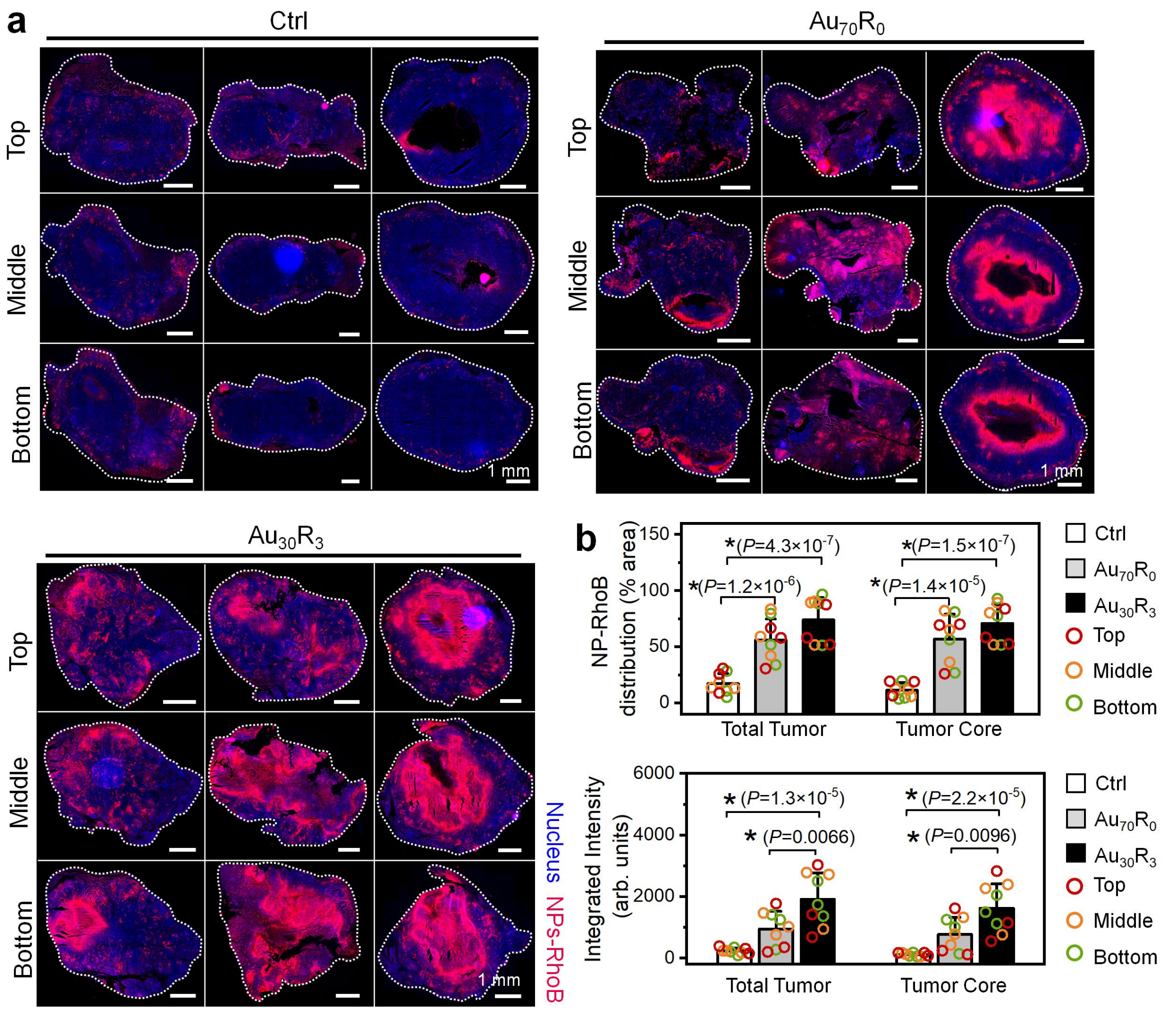

Supplementary Figure 23. NanoEL particles increases vascular leakiness resulting in deeper tumoral penetration in Panc02 orthotopic tumor model. (**a**) Panc02 orthotopic tumor sections revealed that control group showed little penetration of the NPs-RhoB into the interior sections of the tumor while $Au_{70}R_0$ and $Au_{30}R_3$ visually showed significantly higher penetration of NPs-RhoB into the tumor. Scale bar: 1 mm. (**B**) Quantification of the NPs-RhoB coverage area and intensity in the representative images of Panc02 orthotopic tumor sections showed deeper and higher concentration of NPs-RhoB penetration into the core regions of the tumor. Data are mean ± SD, n=9 independent tumor sections, N=3 mice/group, One-way ANOVA Tukey HSD post-hoc test, *significant against control, $P<0.05$. Source data are provided as a Source Data file.

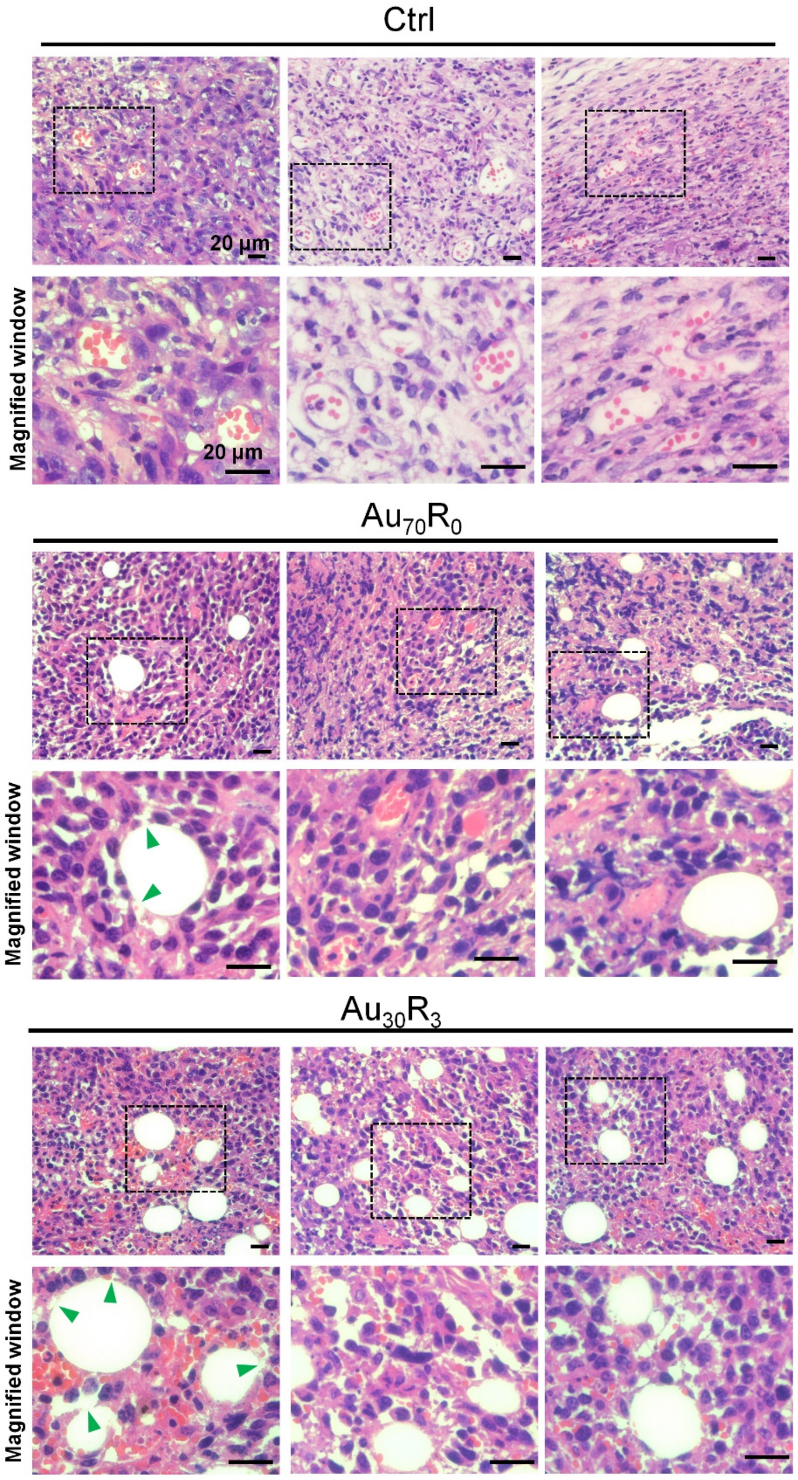

Supplementary Figure 24. NanoEL particles increases occurrence of red blood cells in the interstitial space. Hematoxylin and Eosin (H&E) of the cross section of the 4T1 ectopic tumor in the control group show RBC to remain largely confined to the tumor vasculature, while in Au NPs treated group, more RBC were found in the interstitial spaces and not within the tumor vasculature. In contrast, RBC. Scale bar: 20 μm. The images shown are representative of three independent experiments.

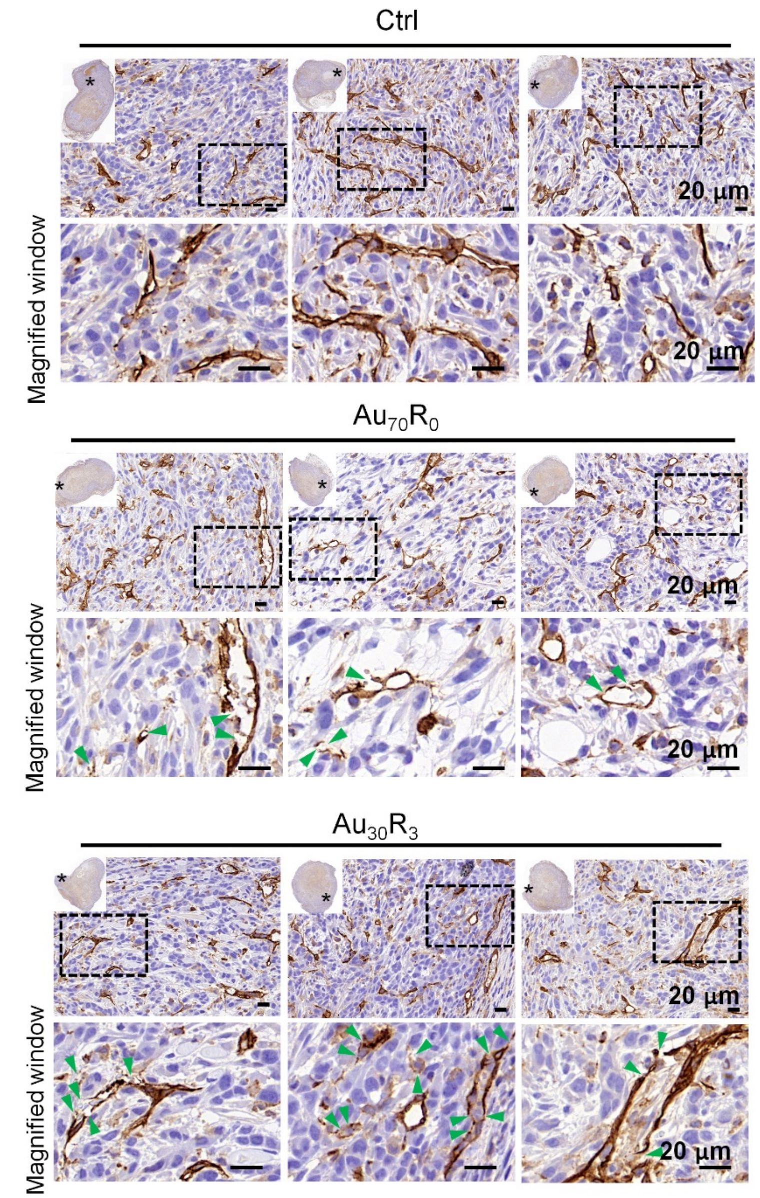

	# Gaps/mm	# Animals	# Vessels	Cross area (mm^2)
Ctrl	3.6 ± 2.8	3	68	0.011
$Au_{70}R_0$	11.8 ± 4.5	3	52	0.014
$Au_{30}R_3$	15.9 ± 4.0	3	60	0.016

Supplementary Figure 25. NanoEL particles increases inter-endothelial gaps. CD31 IHC of ectopic tumor vasculature in Au NPs treated group appears to be distended with occurrences of gaps (green arrows) around the circumferential region of the vasculature. n=3 mice/group. Scale bar: 20 µm. Summary table depicts increase in the gaps detected following Au NPs treatment.

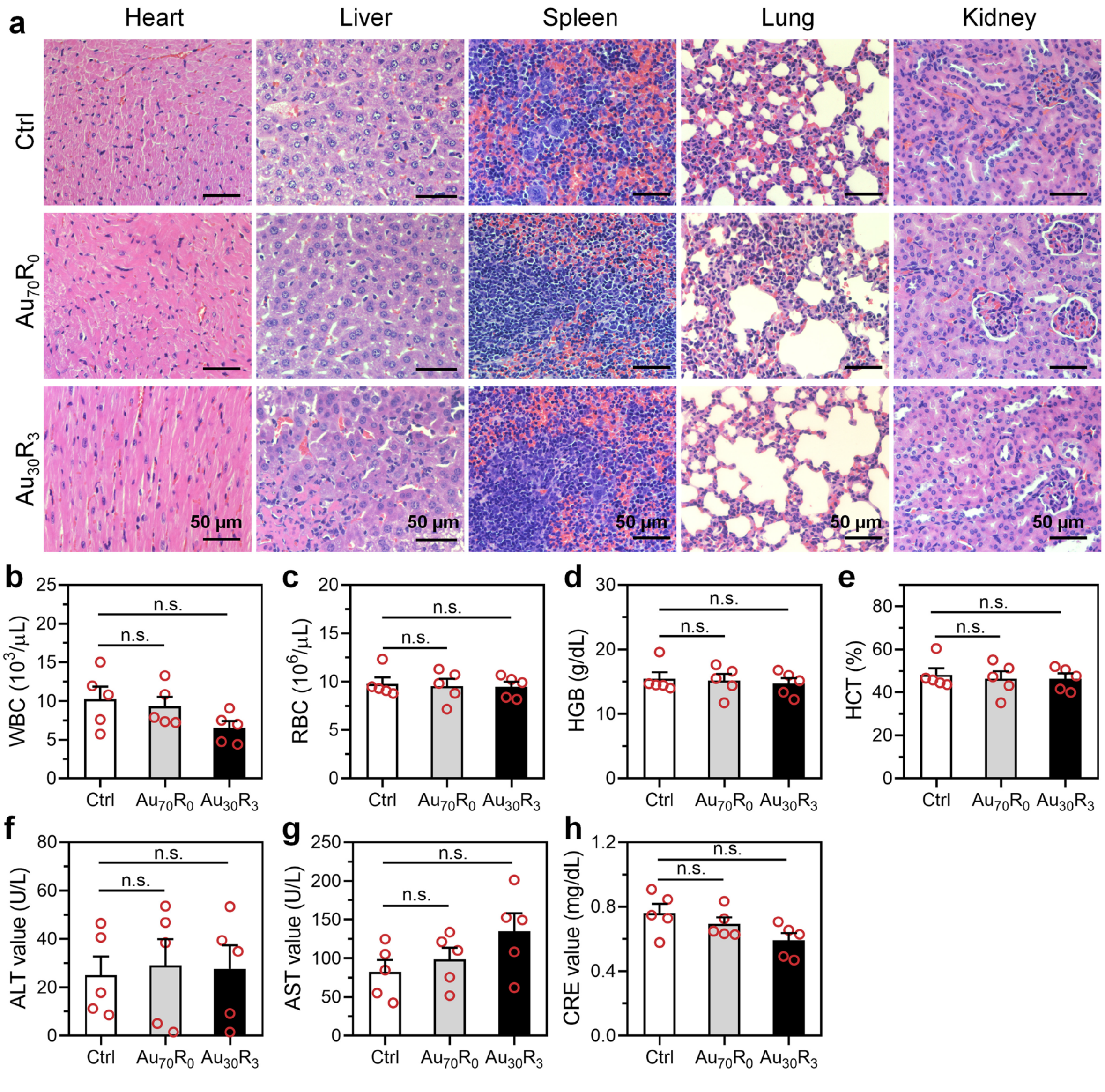

Supplementary Figure 26. Au NPs treatment did not cause any cytotoxic side effects in mice. The mice were intravenously injected with 10 mg/kg $Au_{70}R_0$, $Au_{30}R_3$, or BSA (Ctrl). After 24 h, the blood and normal organs were harvested for analyses. (**a**) H&E images of normal organs showed no perceivable morphological difference between the treatment groups. Scale bar: 50 µm. Additionally, no significant difference was observed on: (**b**) White blood cell (WBC) count. (**c**) Red blood cell (RBC) count. (**d**, **e**) Blood biochemical indices including (**d**) concentration of hemoglobin (HGB) and (**e**) percentage of hematocrit (HCT). n=5 mice/group. Function of liver evaluated using values of (**f**) alanine aminotransferase (ALT), and (**g**) aspartate aminotransferase (AST) in the serum. (**h**) Function of kidney evaluated using creatinine (CRE) in the serum. Data are mean ± SEM, One-way ANOVA Tukey HSD post-hoc test. n=5 mice/group. Source data are provided as a Source Data file.

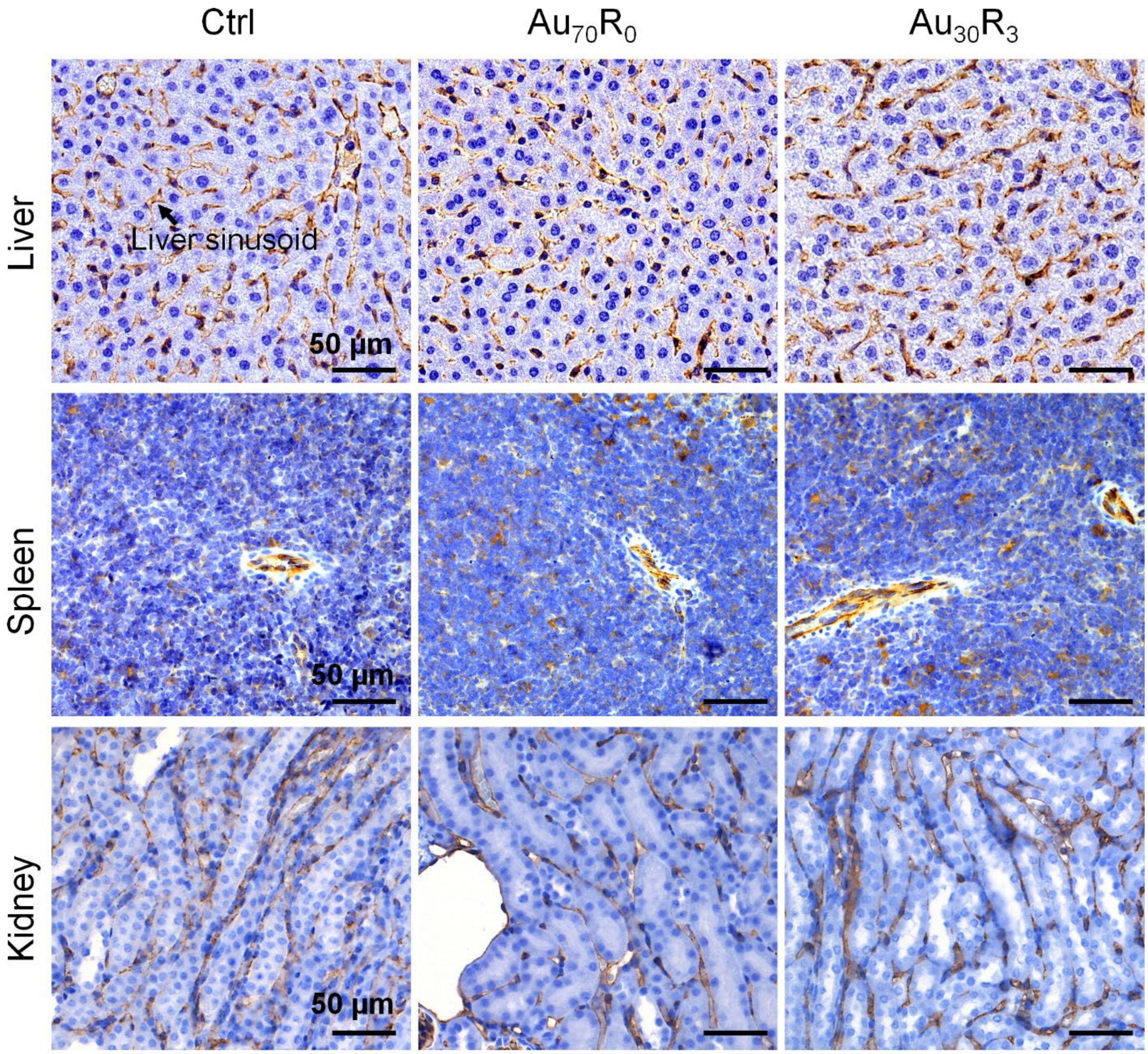

Supplementary Figure 27. Au NPs treatment did not cause any vascular damage in the normal organs. The mice were intravenously injected with 10 mg/kg $Au_{70}R_0$, $Au_{30}R_3$, or BSA (Ctrl). After 24 h, the normal organs (*i.e.*, liver, spleen, and kidney) were harvested for analyses. CD31 IHC images of normal organs vasculature showed no perceivable morphological difference between the treatment groups (n= 3 mice/groups). Scale bar: 50 μm.

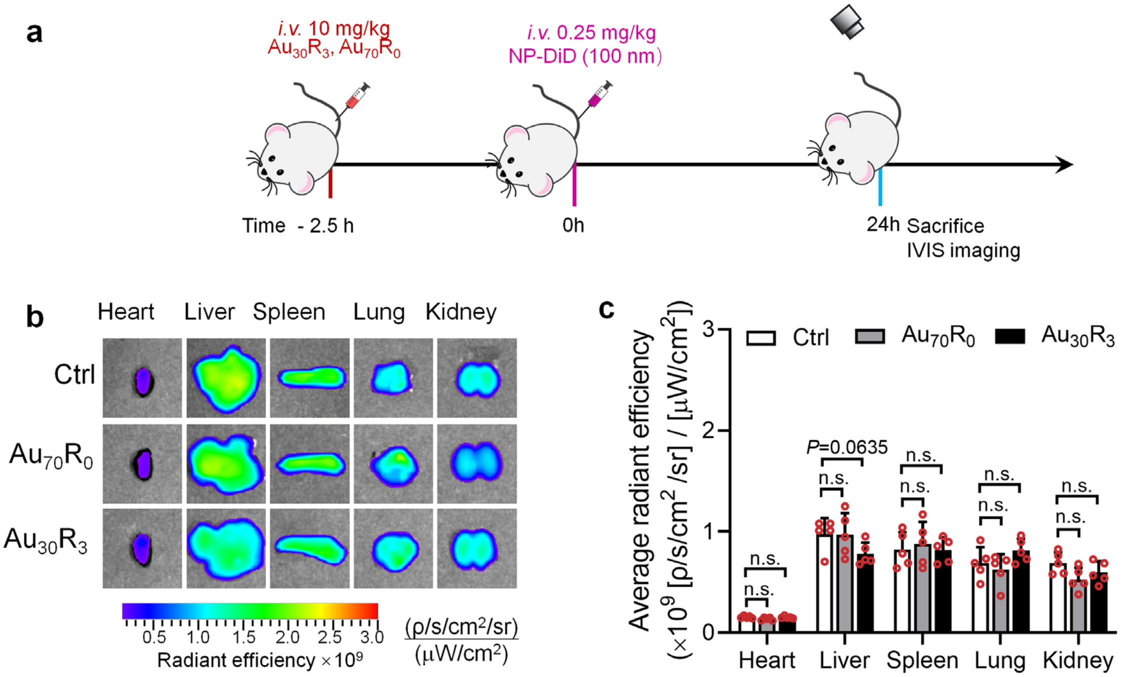

Supplementary Figure 28. NanoEL is not detected in normal tissue. (**a**) Schematic of treatment showing normal mice bearing no tumor (n=5 mice/group) treated with 10 mg/kg $Au_{30}R_3$, $Au_{70}R_0$. BSA (1%) was used as vehicle control, and NPs-DiD (0.25 mg/kg) was used to visualize the organs. (**b**) Representative IVIS images and (**c**) Quantification of the average radiant efficiency of the organs show no discernable change in the DiD accumulation profile between the treatment groups, suggesting Au NPs did not induce leakiness in normal tissue. Data are mean ± SEM, n=5 mice/group. Two-way ANOVA Tukey HSD post-hoc test, *significant against control, $P<0.05$. Source data are provided as a Source Data file.

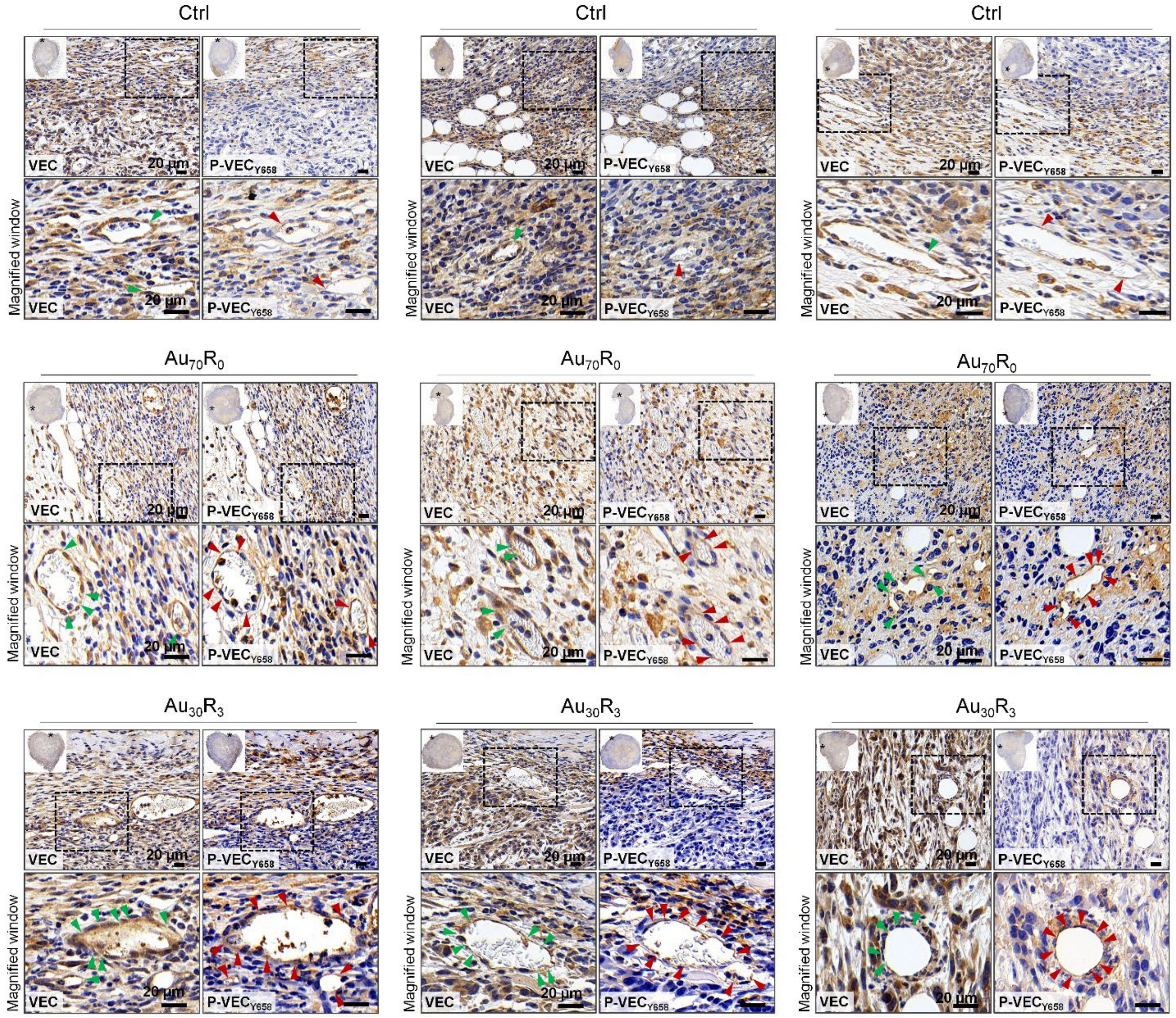

Supplementary Figure 29. Au NPs treatment activates the VE-cadherin signaling in the in vivo setting Bright field images of IHC VE-cadherin (VEC) and phosphorylated VE-cadherin (P-VEC (Y658)) IHC staining on the 4T1 orthotopic tumor vasculature cross sectional. VE-cadherin (VEC) IHC staining of the cross section of the tumor shows reduction of the VEC expressing cells along the perimeter of tumor vasculature of Au NPs treated groups. Occurrence of gaps around the circumferential region of the tumor vasculature is noted with green arrows. Concomitantly, IHC staining shows increased cell expressing phosphorylated VE-cadherin (P-VEC (Y658)) (red arrows), suggesting the activation of VE-cadherin signaling. Scale bar: 20 µm. The images shown are representative of three independent experiments.

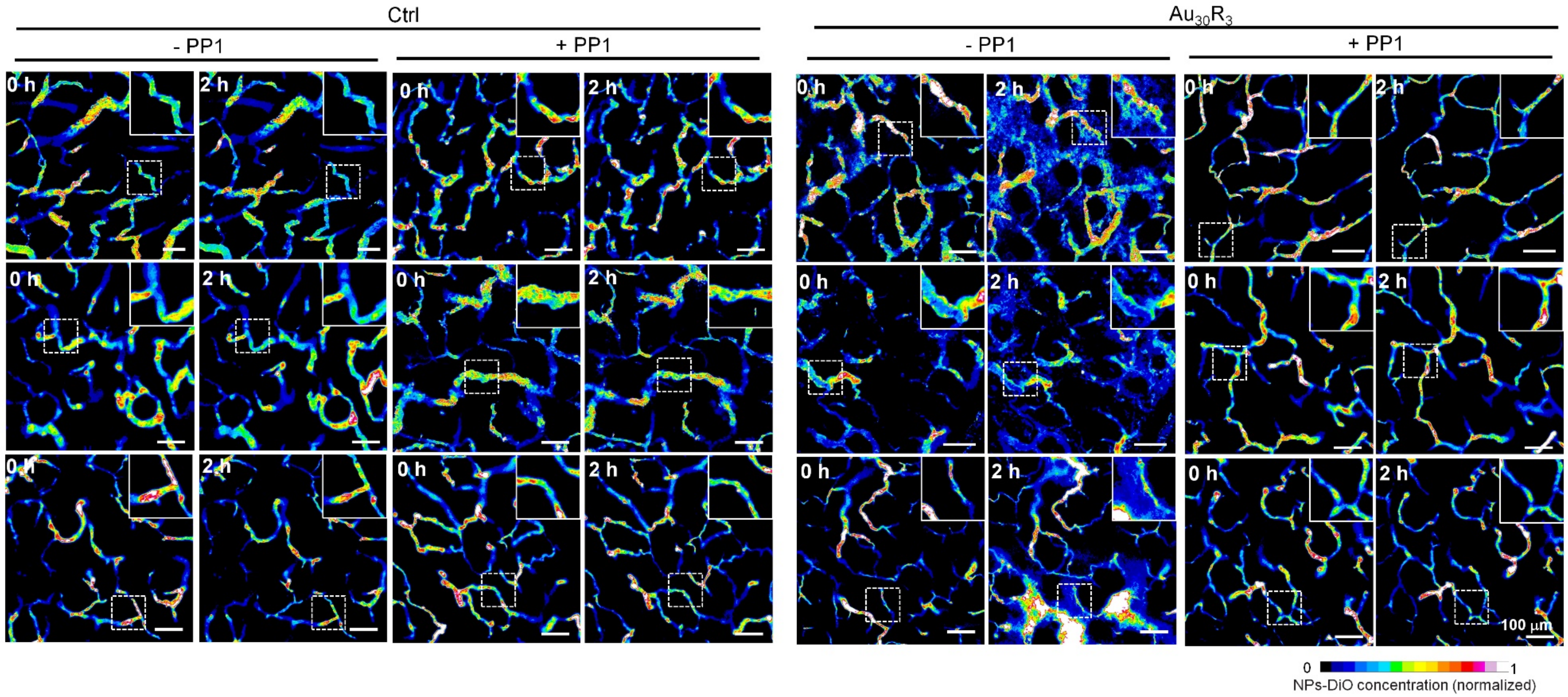

Supplementary Figure 30. Inhibiting VE-cadherin signaling through PP1 treatment reduced NanoEL observed in 4T1 ectopic tumor vasculature. Murine 4T1 breast tumor cells were ectopically implanted on the mouse ear flap (n=3 mice/group). Mice were intravenously injected with PP1 (1.5 mg/kg) 1 h prior being treated either with vehicle control or NanoEL particles, $Au_{30}R_3$ (10 mg/kg). NPs-DiO (~100 nm) were used as common visualization particles, and the *in vivo* leakiness was viewed through intravital imaging (IVM).

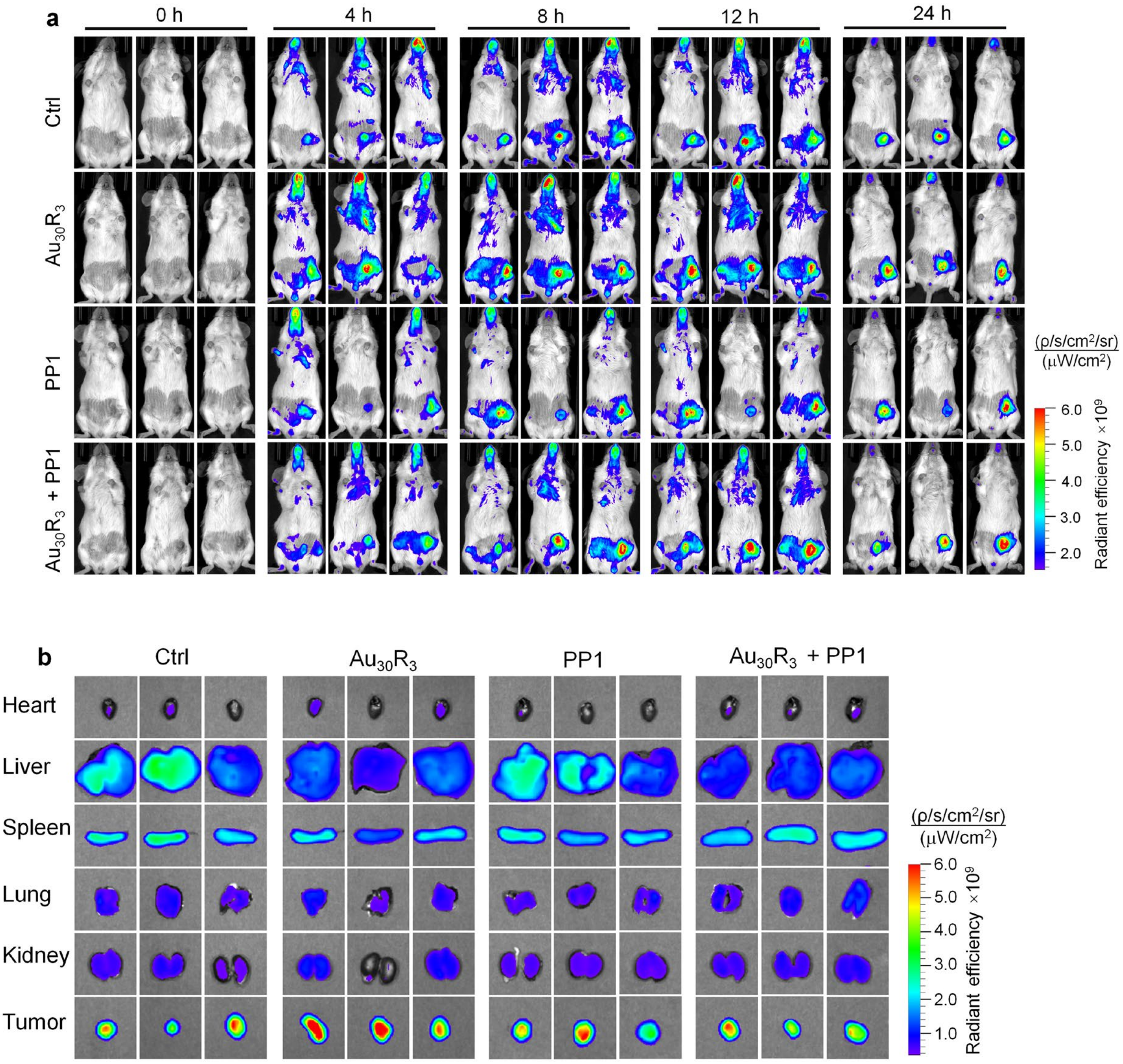

Supplementary Figure 31. VE-cadherin signaling is required in NanoEL assisted particle penetration *in vivo*. Imaging of (**a**) live animal implanted with orthotopic 4T1 tumors (n=3 mice/group) and (**b**) various organs (n=3 mice/group) harvested from the mice receiving $Au_{30}R_3$ NPs (10 mg/kg) in the presence/absence of PP1 treatment (1.5 mg/kg). IVIS imaging system to visualize the accumulation of tracer particles, 100nm NPs-DiD. PP1 was noted to reduce the accumulation of fluorescent particles in the tumor, suggesting the pivotal role of VE-cadherin signaling process in the induction of NanoEL in *in vivo* setting.

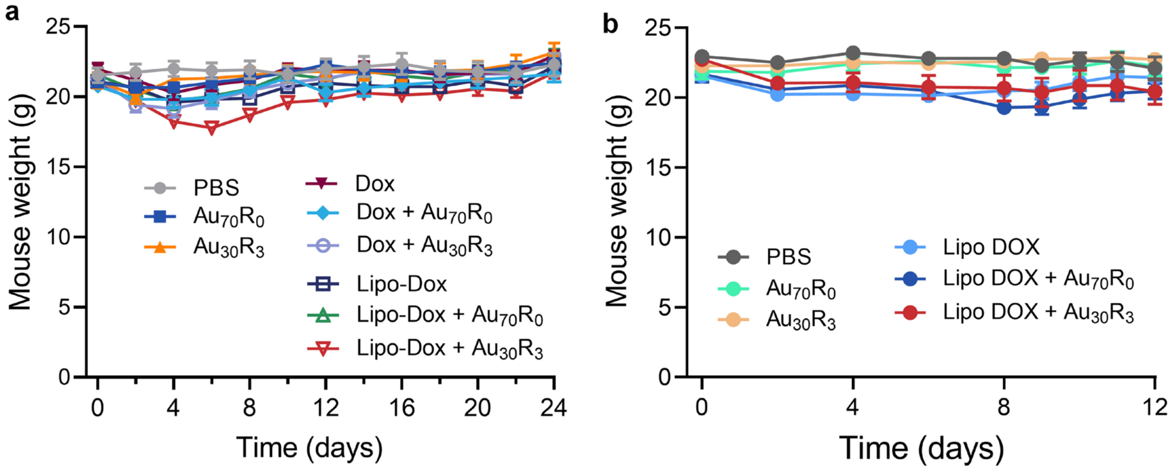

Supplementary Figure 32. No perceivable change on the mice weight in different treatment groups. (**a**) Mice bearing early stage 4T1/luc tumor. The weight of mice (n=7/group) treated with different formulation groups were tracked over the course of the study (24 days). No perceivable weight change was observed over the different treatment groups. Data are mean ± SEM (n=7 mice/group) (**b**) Mice bearing late stage 4T1/luc tumor. The weight of mice (n=6/group) treated with different formulation groups were tracked over the course of the study (12 days). No perceivable weight change was observed over the different treatment groups. Data are mean ± SEM (n=6 mice/group). Source data are provided as a Source Data file.

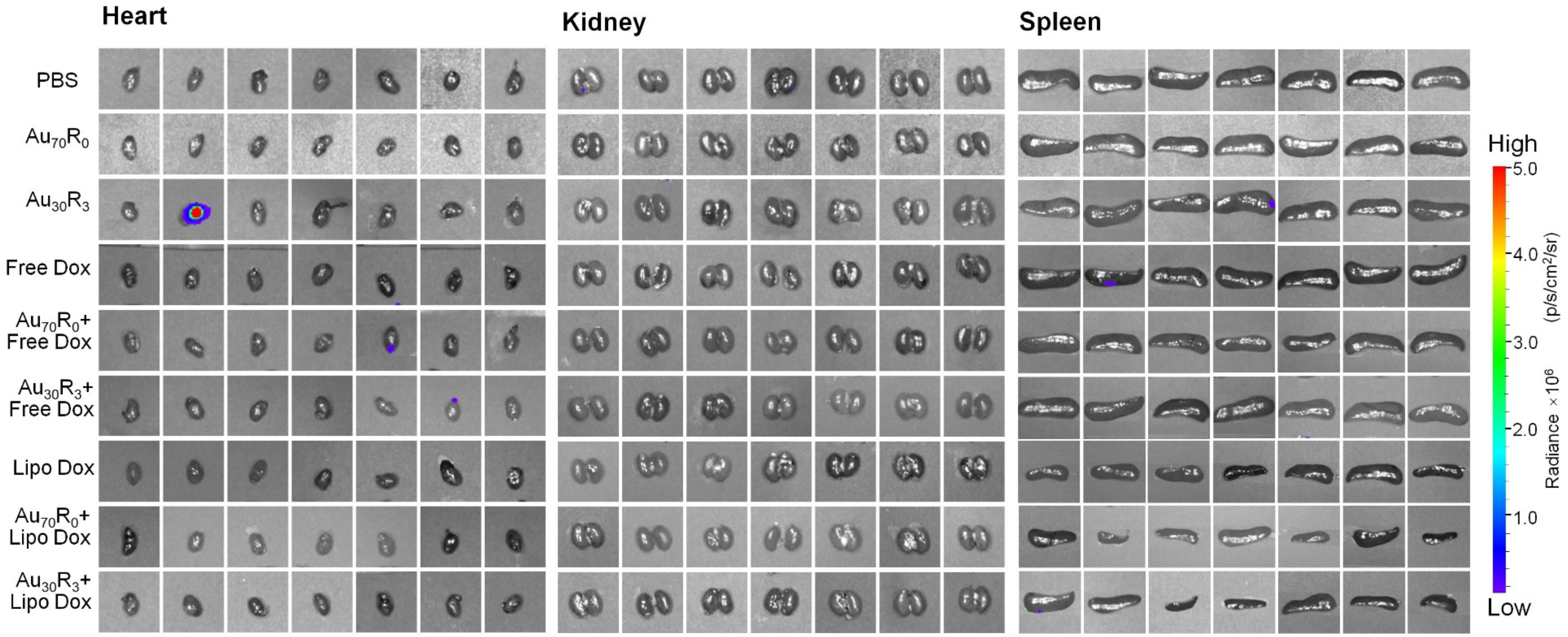

Supplementary Figure 33. NanoEL particles effect on metastasis process. The various organs were scanned for the metastatic 4T1/luc cancer cells using the IVIS imaging system show minimum to no metastasis to the heart, kidney, and spleen.

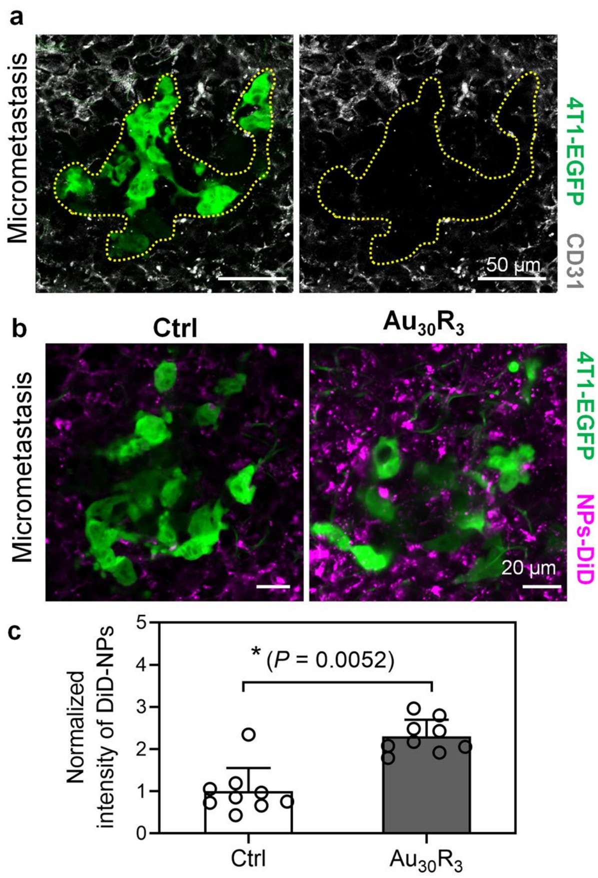

Supplementary Figure 34. NanoEL induction in micrometastasis 4T1 tumor. Mice (n=3 mice/group) were orthotopically implanted with 4T1-EGFP tumor of which it was resected from the primary site when reaching ~500 mm^3. Immediately thereafter the mice received intravenous injection either with vehicle control or NanoEL particles, $Au_{30}R_3$ (10 mg/kg). NPs-DiD tracer and PE-conjugated anti-CD31 were used to visualize the *in vivo* leakiness and tumor neovascularization, respectively. (**a**) Representative IVM images of the lung metastatic secondary tumors. Neovascularization (grey) was not observed on micrometastasis tumor. 4T1 tumor cells (green). Scale bar: 50 μm. (**b**) Representative IVM image and (**c**) quantification of the NPs-DiD tracer intensity show increased leakiness into the interstitial space surrounding micrometastasis in the $Au_{30}R_3$ nanoparticles group. Scale bar: 20 μm. Data are mean ± SD (n=9 ROI from 3 mice/group). Source data are provided as a Source Data file.

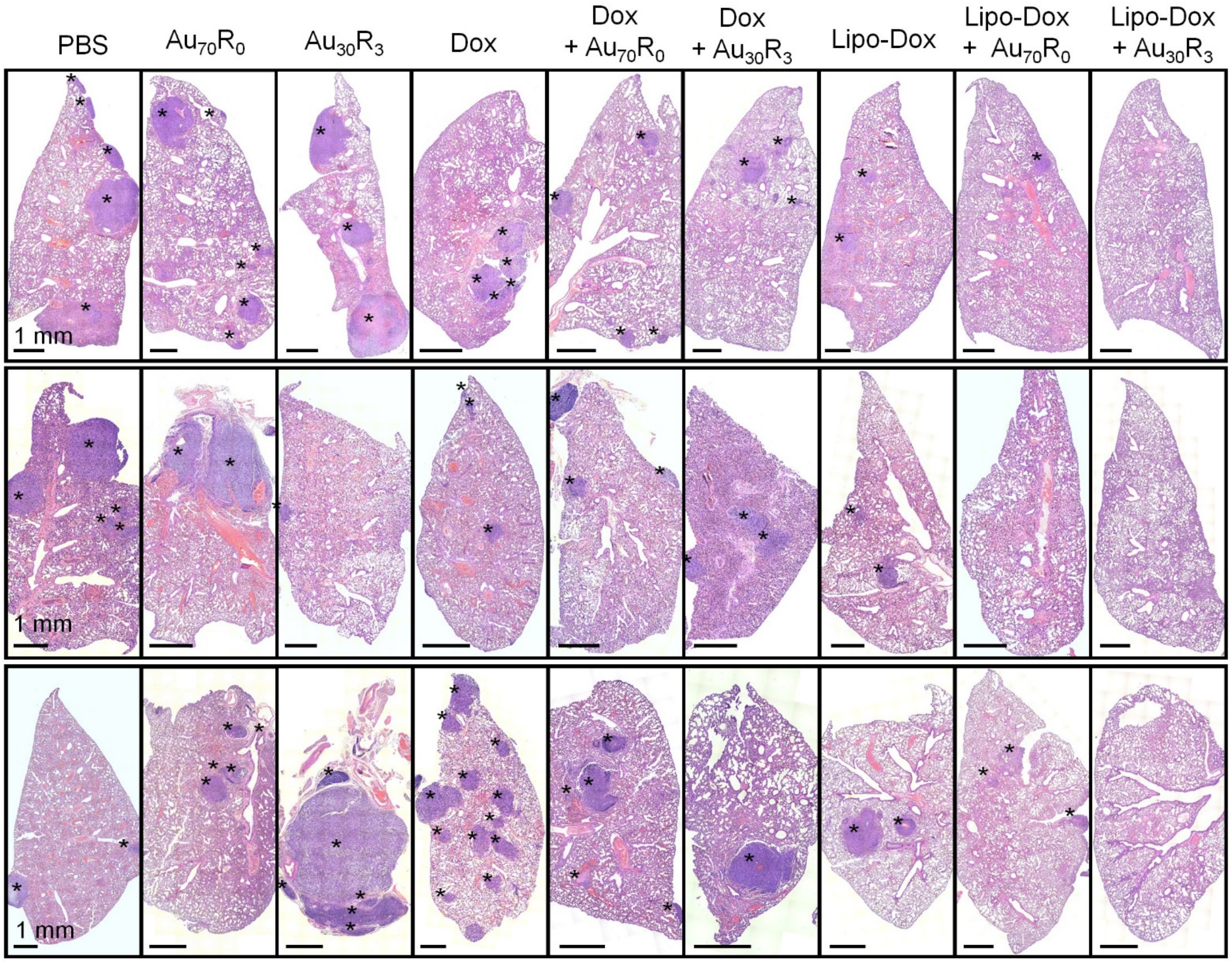

Supplementary Figure S35. NanoEL particles effect on metastasis process. Lung histology sections show cotreatment of Lipo-Dox with NPs inducing NanoEL (i.e, $Au_{70}R_0$ and $Au_{30}R_3$) significantly reduce the metastatic burden in the mice orthotopically implanted with 4T1 breast tumor. Scale bar: 1 mm. The images shown are representative of three independent experiments.

Supplementary Table 1. Summary of Au NPs physiochemical and hydrodynamic properties

Au NPs	Primary size (nm)[a]	Ultrapure water		Complete EndoGRO MV-VEGF medium	
		Hydrodynamic size (nm)[b]	ζ-potential (mV)[b]	Hydrodynamic size (nm)[b]	ζ-potential (mV)[b]
$Au_{30}R_0$	29.9 ± 5.0	29.4 ± 1.9	– 26.6 ± 1.7	44.2 ± 1.7	– 17.6 ± 0.6
$Au_{30}R_1$	31.5 ± 5.8	27.6 ± 2.5	– 28.4 ± 2.1	45.1 ± 1.1	– 17.0 ± 0.8
$Au_{30}R_2$	29.1 ± 6.5	28.8 ± 0.7	– 32.4 ± 1.8	47.0 ± 0.4	– 16.9 ± 0.1
$Au_{30}R_3$	31.6 ± 4.1	27.1 ± 1.6	– 30.0 ± 2.4	43.5 ± 2.0	– 16.3 ± 0.2
$Au_{70}R_0$	73.0 ± 11.1	66.9 ± 2.3	– 29.8 ± 3.0	106.7 ± 3.3	– 15.9 ± 0.1
$Au_{70}R_1$	74.1 ± 7.7	65.5 ± 4.5	– 29.5 ± 0.4	109.7 ± 13.7	– 17.4 ± 1.0
$Au_{70}R_2$	74.9 ± 8.2	63.2 ± 0.2	– 33.1± 1.7	115.3 ± 4.6	– 16.4 ± 0.3
$Au_{70}R_3$	75.7 ± 6.5	69.5 ± 1.5	– 31.7 ± 3.4	105.8 ± 5.6	– 15.6 ± 0.4

Data presented are mean ± SD
[a]Determined from TEM micrographs (n=50) pooled from three independent experiment.
[b]Determined from Dynamic Light Scattering (DLS) analysis (n=3).
Source data are provided as a Source Data file.

Supplementary Table 2. Volumetric centrifugation method in vitro sedimentation, diffusion and dosimetry (VCM-ISDD) estimation of Au NPs deposition fraction and delivered dose on cells grown in Transwell insert (24-well plate) and 96-well plate format. Comparable deposited fraction across these different sized groups of Au NPs over the short time period (0.5 – 1 h) of which the NanoEL induction was initially detected.

**Au NP**	**α (h^{-1})**	**t_{99} (h)**	**t_{90} (h)**	**t_{50} (h)**	**$f_{D,0.5}$**	**$f_{D,1}$**
$Au_{30}R_0$	0.0202	227.5	113.8	34.2	0.056	0.078
$Au_{30}R_1$	0.0201	229.2	114.6	34.5	0.055	0.078
$Au_{30}R_2$	0.0198	232.1	116	34.9	0.054	0.054
$Au_{30}R_3$	0.0203	227.4	113.7	34.2	0.056	0.079
$Au_{70}R_0$	0.0463	99.4	49.7	15	0.064	0.095
$Au_{70}R_1$	0.0466	98.9	49.4	14.9	0.064	0.094
$Au_{70}R_2$	0.0518	88.9	44.4	13.4	0.064	0.094
$Au_{70}R_3$	0.0444	103.7	51.8	15.6	0.064	0.094

α, Specific Au NPs deposition fraction constant in complete EndoGRO medium; t_{99}, Au NPs exposure time to achieve 99% Au NPs fraction deposition; t_{90}, Au NPs exposure time to achieve 90% Au NPs fraction deposition; t_{50}, Au NPs exposure time to achieve 50% Au NPs fraction deposition; $f_{D,0.5}$, Au NPs deposition fraction at 30 min exposure; $f_{D,1}$, Au NPs deposition fraction at 1 h exposure.
Source data are provided as a Source Data file.

Supplementary Table 3. Volumetric centrifugation method in vitro sedimentation, diffusion and dosimetry (VCM-ISDD) estimation of Au NPs deposition fraction and delivered dose on cells grown in 6-well plate format. Comparable deposited fraction across these different sized groups of Au NPs over the short time period (0.5 – 1 h) of which the NanoEL induction was initially detected

**Au NPs**	**α (h^{-1})**	**t_{99} (h)**	**t_{90} (h)**	**t_{50} (h)**	**$f_{D,0.5}$**	**$f_{D,1}$**
$Au_{30}R_0$	0.0242	189.9	95	28.6	0.064	0.091
$Au_{30}R_1$	0.024	191.7	95.9	28.9	0.063	0.09
$Au_{30}R_2$	0.0236	194.9	97.4	29.3	0.062	0.062
$Au_{30}R_3$	0.0243	189.2	94.6	28.5	0.065	0.091
$Au_{70}R_0$	0.0577	79.8	39.9	12	0.073	0.108
$Au_{70}R_1$	0.0581	79.3	39.6	11.9	0.073	0.108
$Au_{70}R_2$	0.0649	71	35.5	10.7	0.074	0.108
$Au_{70}R_3$	0.0553	83.3	41.7	12.5	0.072	0.107

α, Specific Au NPs deposition fraction constant in complete EndoGRO medium; t_{99}, Au NPs exposure time to achieve 99% Au NPs fraction deposition; t_{90}, Au NPs exposure time to achieve 90% Au NPs fraction deposition; t_{50}, Au NPs exposure time to achieve 50% Au NPs fraction deposition; $f_{D,0.5}$, Au NPs deposition fraction at 30 min exposure; $f_{D,1}$, Au NPs deposition fraction at 1 h exposure.
Source data are provided as a Source Data file.

Supplementary Table 4. Volumetric centrifugation method in vitro sedimentation, diffusion and dosimetry (VCM-ISDD) estimation of Au NPs deposition fraction and delivered dose on cells grown in 6 cm dish format. Comparable deposited fraction across these different sized groups of Au NPs over the short time period (0.5 – 1 h) of which the NanoEL induction was initially detected

**Au NPs**	**α (h^{-1})**	**t_{99} (h)**	**t_{90} (h)**	**t_{50} (h)**	**$f_{D,0.5}$**	**$f_{D,1}$**
$Au_{30}R_0$	0.0298	154.6	77.3	23.3	0.0747	0.1055
$Au_{30}R_1$	0.0294	156.4	78.2	23.5	0.074	0.1045
$Au_{30}R_2$	0.0288	159.7	79.9	24	0.0725	0.1025
$Au_{30}R_3$	0.03	153.5	76.8	23.1	0.0753	0.1063
$Au_{70}R_0$	0.0724	63.6	31.8	9.6	0.0839	0.1245
$Au_{70}R_1$	0.0729	63.2	31.6	9.5	0.0835	0.1239
$Au_{70}R_2$	0.0815	56.5	28.2	8.5	0.0847	0.1249
$Au_{70}R_3$	0.0693	66.5	33.2	10	0.0828	0.1233

α, Specific Au NPs deposition fraction constant in complete EndoGRO medium; t_{99}, Au NPs exposure time to achieve 99% Au NPs fraction deposition; t_{90}, Au NPs exposure time to achieve 90% Au NPs fraction deposition; t_{50}, Au NPs exposure time to achieve 50% Au NPs fraction deposition; $f_{D,0.5}$, Au NPs deposition fraction at 30 min exposure; $f_{D,1}$, Au NPs deposition fraction at 1 h exposure.
Source data are provided as a Source Data file.
