# Supplementary material for: Engineering tumoral vascular leakiness with gold nanoparticles
Source: Nat Commun. 2023 Jul 17;14:4269. doi: 10.1038/s41467-023-40015-4 (PMC10352264; doi:10.1038/s41467-023-40015-4)
Supplement: Supplementary file 3 — Description to Additional Supplementary Information [file 41467_2023_40015_MOESM3_ESM.pdf]

Description of Additional Supplementary Files

Supplementary Movie 1- related to the IVM imaging of tumor vasculature implanted into the ear flaps (Control group)

Supplementary Movie 2- related to the IVM imaging of tumor vasculature implanted into the ear flaps ($Au_{30}R_3$ group)
