# Supplementary material for: Engineering tumoral vascular leakiness with gold nanoparticles
Source: Nat Commun. 2023 Jul 17;14:4269. doi: 10.1038/s41467-023-40015-4 (PMC10352264; doi:10.1038/s41467-023-40015-4)
Supplement: Supplementary file 6 — Reporting Summary [file 41467_2023_40015_MOESM6_ESM.pdf]

nature portfolio

Corresponding author(s): David Tai Leong, Magdiel Inggrid Setyawati, Yucai Wang

Last updated by author(s): May 3, 2023

Reporting Summary

Nature Portfolio wishes to improve the reproducibility of the work that we publish. This form provides structure for consistency and transparency in reporting. For further information on Nature Portfolio policies, see our Editorial Policies and the Editorial Policy Checklist.

Statistics

For all statistical analyses, confirm that the following items are present in the figure legend, table legend, main text, or Methods section.

n/a	Confirmed	
☐	☒	The exact sample size (*n*) for each experimental group/condition, given as a discrete number and unit of measurement
☐	☒	A statement on whether measurements were taken from distinct samples or whether the same sample was measured repeatedly
☐	☒	The statistical test(s) used AND whether they are one- or two-sided<br>*Only common tests should be described solely by name; describe more complex techniques in the Methods section.*
☒	☐	A description of all covariates tested
☒	☐	A description of any assumptions or corrections, such as tests of normality and adjustment for multiple comparisons
☐	☒	A full description of the statistical parameters including central tendency (e.g. means) or other basic estimates (e.g. regression coefficient) AND variation (e.g. standard deviation) or associated estimates of uncertainty (e.g. confidence intervals)
☐	☒	For null hypothesis testing, the test statistic (e.g. *F*, *t*, *r*) with confidence intervals, effect sizes, degrees of freedom and *P* value noted<br>*Give P values as exact values whenever suitable.*
☒	☐	For Bayesian analysis, information on the choice of priors and Markov chain Monte Carlo settings
☒	☐	For hierarchical and complex designs, identification of the appropriate level for tests and full reporting of outcomes
☒	☐	Estimates of effect sizes (e.g. Cohen's *d*, Pearson's *r*), indicating how they were calculated

Our web collection on statistics for biologists contains articles on many of the points above.

Software and code

Policy information about availability of computer code

Data collection	Intravital microscopy images were acquired with a Nikon Ti2 confocal laser scanning microscope system attached to an upright ECLIPSE Ni-E equipped using the software NIS-Elements AR (v. 5.20.00). IVIS images were collected using Living Image software (v. 4.5). Images of the tissue sections were acquired with the automated quantitative microscopy-based image analysis system TissueFAXS PLUS (v. 4.2).
Data analysis	Intravital microscopy images and fluorescent images were processed with FIJI (v. 2.1.0). Data analysis and plotting were done with GraphPad Prism (v. 8.0) or Origin (v. 9.5). IVIS analysis were analyzed using Living Image software (v. 4.5). No custom code/algorithm were used.

For manuscripts utilizing custom algorithms or software that are central to the research but not yet described in published literature, software must be made available to editors and reviewers. We strongly encourage code deposition in a community repository (e.g. GitHub). See the Nature Portfolio guidelines for submitting code & software for further information.

Data

Policy information about availability of data

All manuscripts must include a data availability statement. This statement should provide the following information, where applicable:

- Accession codes, unique identifiers, or web links for publicly available datasets
- A description of any restrictions on data availability
- For clinical datasets or third party data, please ensure that the statement adheres to our policy

Data are available within the Article, Supplementary Information or Source Data file. Source data are provided with this paper.

Human research participants

Policy information about studies involving human research participants and Sex and Gender in Research.

Reporting on sex and gender	N/A
Population characteristics	N/A
Recruitment	N/A
Ethics oversight	N/A

Note that full information on the approval of the study protocol must also be provided in the manuscript.

Field-specific reporting

Please select the one below that is the best fit for your research. If you are not sure, read the appropriate sections before making your selection.

☒ Life sciences ☐ Behavioural & social sciences ☐ Ecological, evolutionary & environmental sciences

For a reference copy of the document with all sections, see nature.com/documents/nr-reporting-summary-flat.pdf

Life sciences study design

All studies must disclose on these points even when the disclosure is negative.

Sample size	The appropriate sample size was determined based on previously published literature on animal study (Nature Communications 2020, 11, 1126) and in vitro work (Nature Communication 2013, 4 , 1673). All our in vitro experiments described in this paper were performed with n = 3-5. Three independent repeats is required to evaluate statistical significance. The in vivo efficacy studies were performed with 3-7 mice per group in both nanoparticles treated and control groups while adhering to our approved University of Science and Technology of China Animal Care and Use Committee standard protocols and minimizing the number of animals to adhere to ethical considerations without compromising on statistical validity. Details regarding sample sizes and statistical tests of all experiments are provided in the Methods section and figure captions.
Data exclusions	No data were excluded from the analyses.
Replication	The in vitro experiments were repeated for 3 times, and the results were consistent with each other. For the in vivo work, each group contains 3-7 mice. All experiments were repeated and findings were consistent.
Randomization	Randomization was relevant in the mice experiments as we are not asking age and sex dependent questions in this study. Thus the mice cohort are all aged matched and sex matched before mice randomization to decrease (if any) age and sex influences. For all other experiments, cells/samples were randomly assigned into groups.
Blinding	The sub team analyzing the mouse data were distinct from the sub team working on the mouse experiments itself and are blinded from the groups. Remaining studies were not blinded as the individual(s) performing the experiment/analysis were also responsible for preparing the samples/studies. Instead, the experiments were replicated and/or repeated as described above with consistent results.

Reporting for specific materials, systems and methods

We require information from authors about some types of materials, experimental systems and methods used in many studies. Here, indicate whether each material, system or method listed is relevant to your study. If you are not sure if a list item applies to your research, read the appropriate section before selecting a response.

Materials & experimental systems

n/a	Involved in the study	
☐	☒	Antibodies
☐	☒	Eukaryotic cell lines
☒	☐	Palaeontology and archaeology
☐	☒	Animals and other organisms
☒	☐	Clinical data
☒	☐	Dual use research of concern

Methods

n/a	Involved in the study	
☒	☐	ChIP-seq
☒	☐	Flow cytometry
☒	☐	MRI-based neuroimaging

Antibodies

Antibodies used	Phospho VE-cadherin (Y658) Rabbit Antibody (Invitrogen, #441144G); Phospho VE-cadherin (Y731) Rabbit Antibody (Invitrogen, #441145G); VE-cadherin Rabbit Antibody (Clone D87F2, Cell Signaling Technology, #2500); β-catenin Rabbit Antibody (Clone H-102, Santa Cruz Biotechnology, #sc-7199); GAPDH Rabbit Antibody (Clone 14C10, Cell Signaling Technology, #2118); HRP-conjugated anti rabbit IgG (Santa Cruz Biotechnology, #sc-2004); Alexa Fluor 647-conjugated anti-rabbit IgG (Life Technologies, #A21443); AlexaFluor 488-phalloidin stain (Life Technologies #A12379); CD31 antibody (Clone D8V9E, Cell Signaling Technology, #77699); VE-Cadherin antibody (Abcam, #ab33168); PE-conjugated anti-CD31 antibody (Clone 390, Biolegend, #102408).
Validation	Antibodies used were commercially available and all antibodies were validated by manufacturers, with related data shown on the manufacturer website:<br>1. Phospho VE-cadherin (Y658) Rabbit Antibody (Invitrogen, #441144G)<br>https://www.thermofisher.com/antibody/product/Phospho-VE-cadherin-Tyr658-Antibody-Polyclonal/44-1144G<br>2. Phospho VE-cadherin (Y731) Rabbit Antibody (Invitrogen, #441145G)<br>https://www.thermofisher.com/antibody/product/Phospho-VE-cadherin-Tyr731-Antibody-Polyclonal/44-1145G<br>3. VE-cadherin Rabbit Antibody (Clone D87F2, Cell Signaling Technology, #2500)<br>https://www.cellsignal.com/products/primary-antibodies/ve-cadherin-d87f2-xp-rabbit-mab/2500?site-search-type=Products&N=4294956287&Ntt=ve+cadherin&fromPage=plp<br>4. β-catenin Rabbit Antibody (Clone H-102, Santa Cruz Biotechnology, #sc-7199)<br>https://www.scbt.com/p/beta-catenin-antibody-h-102?requestFrom=search<br>5. GAPDH Rabbit Antibody (Clone 14C10, Cell Signaling Technology, #2118)<br>https://www.cellsignal.com/products/primary-antibodies/gapdh-14c10-rabbit-mab/2118?site-search-type=Products&N=4294956287&Ntt=gapdh&fromPage=plp<br>6. HRP-conjugated anti rabbit IgG (Santa Cruz Biotechnology, #sc-2004)<br>https://www.scbt.com/p/goat-anti-rabbit-igg-hrp<br>7. Alexa Fluor 647-conjugated anti-rabbit IgG (Life Technologies, #A21443)<br>https://www.thermofisher.com/antibody/product/Chicken-anti-Rabbit-IgG-H-L-Cross-Adsorbed-Secondary-Antibody-Polyclonal/A-21443<br>8. AlexaFluor 488-phalloidin stain (Life Technologies #A12379)<br>https://www.thermofisher.com/order/catalog/product/A12379<br>9. CD31 antibody (Clone D8V9E, Cell Signaling Technology, #77699)<br>https://www.cellsignal.com/products/primary-antibodies/cd31-pecam-1-d8v9e-xp-rabbit-mab/77699<br>10. VE-Cadherin antibody (Abcam, #ab33168)<br>https://www.abcam.com/products/primary-antibodies/ve-cadherin-antibody-intercellular-junction-marker-ab33168.html<br>11. PE-conjugated anti-CD31 antibody (Biolegend, #102408)<br>https://www.biolegend.com/en-us/products/pe-anti-mouse-cd31-antibody-122?GroupID=BLG1566

Eukaryotic cell lines

Policy information about cell lines and Sex and Gender in Research

Cell line source(s)	Primary human microvascular endothelial cells (HMVEC, Thermoscientific, USA, Cat# C-010-5C), mouse breast cancer cell lines 4T1 (ATCC, USA, Cat# CRL-2539) and 4T1/luc (ATCC, USA, Cat# CRL-2539-LUC2), murine colon cancer CT26 cells (ATCC, USA, Cat# CRL-2638), and murine pancreatic cancer Panc02 (provided by Dr Jun Wang (South China University of Technology)) were used in this study.
Authentication	We did not authenticate the cell lines because they were all from commercial sources and they have their own individual companies' quality control policies.
Mycoplasma contamination	All cell lines were tested negative for mycoplasma contamination.
Commonly misidentified lines<br>(See ICLAC register)	No commonly misidentified cell lines were used.

Animals and other research organisms

Policy information about studies involving animals; ARRIVE guidelines recommended for reporting animal research, and Sex and Gender in Research

Laboratory animals	Female BALB/c mice and male C57BL/6 mice were purchased from Beijing Vital River Laboratory Animal Technology. Mice aged between 6 weeks and 8 weeks were used. Animals were maintained at 21±1°C, in 40% to 70% humidity, and with a 12 hours light/dark cycle (from 8 a.m. to 8 p.m.)
Wild animals	This study did not involve wild animals.
Reporting on sex	We selected mice of the appropriate sex according to the requirements of the tumor model and did not specifically factor in sex as a variable in our experimental design. A female mouse model was employed for 4T1 breast cancer and CT26 colon cancer while a male mouse model was used to establish Panc02 pancreatic cancer model.
Field-collected samples	This study did not involve field-collected samples.

Ethics oversight	All animal studies were approved by the institutional review board at University of Science and Technology of China(Approval ID: USTCACUC1801006).

Note that full information on the approval of the study protocol must also be provided in the manuscript.
